# Engineering Photoswitching Dynamics in 3D Photochromic Metal–Organic Frameworks through a Metal–Organic Polyhedron Design

**DOI:** 10.1021/jacs.4c17203

**Published:** 2025-02-25

**Authors:** Eunji Jin, Volodymyr Bon, Shubhajit Das, A. D. Dinga Wonanke, Martin Etter, Martin A. Karlsen, Ankita De, Nadine Bönisch, Thomas Heine, Stefan Kaskel

**Affiliations:** † Chair of Inorganic Chemistry I, Faculty of Chemistry and Food Chemistry, Technische Universität Dresden, Bergstraße 66, 01069 Dresden, Germany; ‡ Chair of Theoretical Chemistry, Faculty of Chemistry and Food Chemistry, Technische Universität Dresden, Bergstraße 66c, 01069 Dresden, Germany; § P02.1 Beamline, PETRA III, Deutsches Elektronen-Synchrotron DESY, Notkestraße 85, 22607 Hamburg, Germany; ∥ Institute of Resource Ecology, Helmholtz Zentrum Dresden-Rossendorf, Permoserstraße 15, 04318 Leipzig, Germany; ⊥ Department of Chemistry, Yonsei University, Seodaemun-gu, Seoul 120-749, Republic of Korea

## Abstract

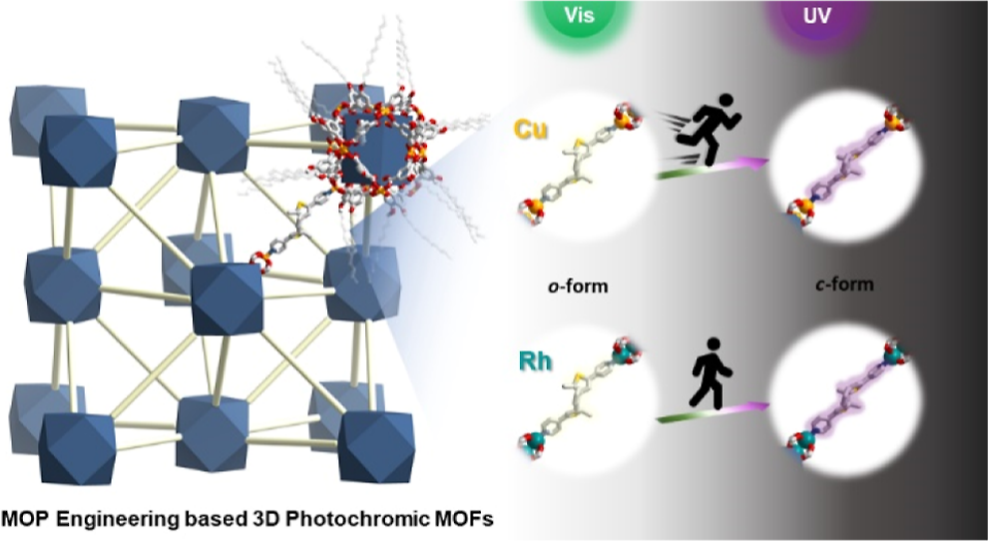

Metal–organic polyhedra (MOPs) are versatile supramolecular building blocks for the design of highly porous frameworks by reticular assembly because of their diverse geometries, multiple degrees of freedom regarding functionalization, and accessible metal sites. Lipophilic functionalization is demonstrated to enable the rational assembly and crystallization with photoactive N-donor ligands in an aliphatic solvent to achieve multiaxially aligned photoresponsive diarylethene (DTE) moieties in 3D frameworks (DUT-210(M), M = Cu and Rh) featuring cooperative switchability. Combined experimental and theoretical investigations based on in situ PXRD, UV–vis spectroscopy, and density functional theory calculations demonstrate deliberate kinetic engineering of photoswitchability based on variations in metal–ligand bond strengths. The novel porous frameworks are an important step toward the knowledge-based development of photon-driven motors, actuators, and release systems.

## Introduction

Beyond static 3D porous frameworks,^1−3^ 4D metal–organic frameworks (MOFs) emphasize the temporal evolution of frameworks, allowing materials to adapt over time, adding a novel functionality to traditional MOFs in recent decades.^4−6^ Their ability for switching kinetics between different structural states based on stimuli such as light, temperature, and pressure has been highlighted for potential applications like sensing, catalysis, and energy storage where time-dependent performance is critical.^7,8^ Specifically, the light-induced photoisomerization kinetics and electron-transfer properties of MOFs have been investigated recently.^9−13^ Photochromism is characterized by either the reversible structural transformation of a chemical species or the photoinduced electron transfer from photoactive molecules, both initiated by the absorption of electromagnetic radiation within defined wavelengths, primarily in the UV/vis/NIR range.^14^ These phenomena hold great potential for various photochromic applications, including light-induced switchable adsorption, separation, electronic sensing, and catalyst, where dynamic electronic properties play a critical role.^15−24^ The archetypical photochromic moieties are diarylethene (DTE), azobenzene, stilbene, and spiropyran.^18^ Photochromic moieties have been incorporated as guest molecules, pendant groups of the components,^9^ and as a backbone of MOFs.^10,11,19,25,26^ To embed photochromic molecules as structural backbones, the pillared layer MOFs have emerged as ideal candidates due to the facile incorporation of photochromic moieties through postsynthetic modification.^27−30^ While 3D photochromic MOFs incorporating photochromic moieties as part of their backbone have recently been studied, the use of rigid ditopic linkers embedding the photochromic component within the framework has made it challenging to achieve significant photoactive responsivity in the 3D structure, especially if flexibility within the framework is not permitted.^31−33^ Furthermore, the understanding and tuning of the kinetics of photoisomerization are challenging tasks that involve overcoming energy barriers, accommodating molecular movements, and addressing cooperative phenomena.

Metal–organic polyhedra (MOPs), assembled from organic linkers and metal nodes whose rational design is based on an elaborated strategy, assisted by topological guidance,^34−38^ have emerged as important cases for discrete nanocages as well as supramolecular building blocks (SBBs) to build extended 3D MOFs.^39−45^ Especially, the surface chemistry of MOPs, which induces the effect of solution processability, is an important factor.^46,47^ The solution processability, a unique feature of MOPs, has enabled the development of MOFs by altering the topology of established 3D MOFs due to extended and connected geometry of MOPs. For example, one of the iconic MOPs, cuboctahedral shaped MOPs (**cuo**-MOPs),^48,49^ is constructed from paddlewheel nodes and functionalized benzenedicarboxylic acid (*m*BDC) linkers coordinated with N-donor bidentate ligand of varying lengths, ranging from 2.7 to 11.5 Å. These structures form 3D MOFs with an **fcu-a** topology.^42−45^ Moreover, they often incorporate a nonlinear N-donor ligand, which facilitates the formation of supramolecular polymers.^50−52^ However, building crystalline MOFs with longer bidentate ligands over 11.5 Å from the MOPs was hampered by the lack of mechanical stability and challenges in desolvation.

**Figure 1:**
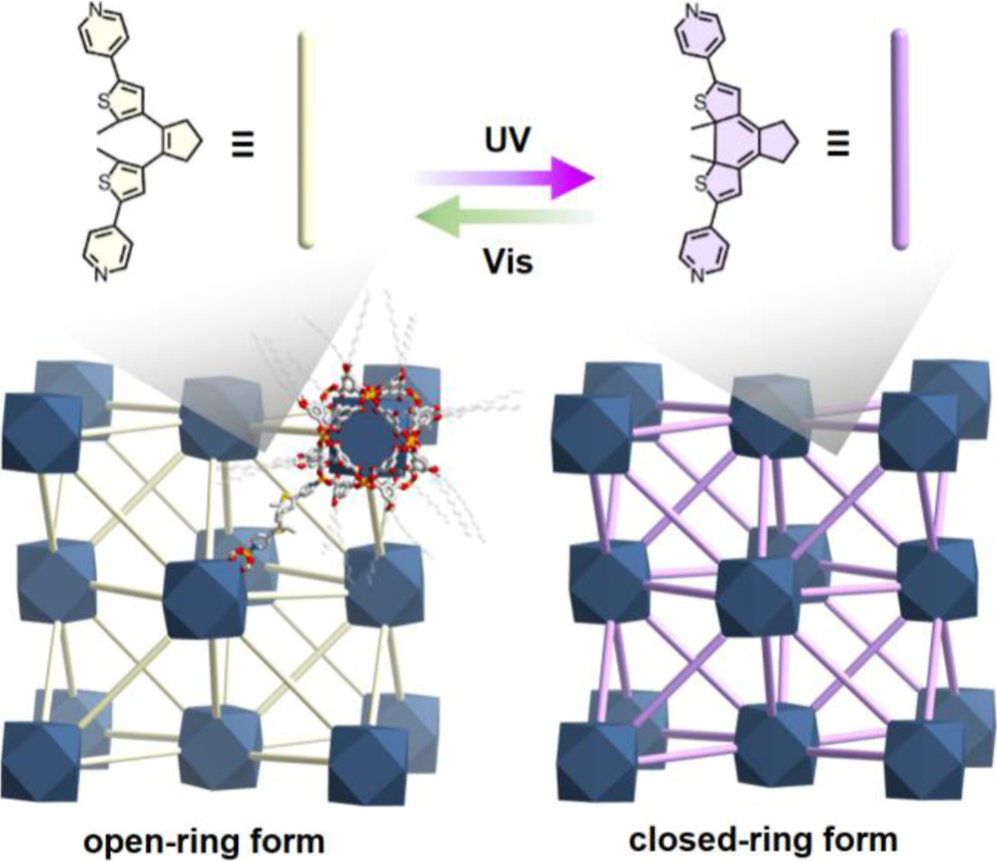
Schematic representation of isotropic photochromic behavior of new pillared 3D MOFs based on MOP engineering.

Herein, we report two new photochromic 3D MOFs, DUT-210(Cu) and DUT-210(Rh) (DUT = Dresden University of Technology), constructed by deliberately connecting MOPs through an N-donor bidentate ligand with a DTE core (Figure 1). These frameworks represent the first ever crystalline 3D photochromic MOFs formed by assembling MOPs with a long photochromic bidentate ligand exceeding 12 Å featuring cooperative switchability. Moreover, DUT-210(Rh) is the first crystalline framework constructed from Rh-based MOPs. A key strategy for the integration of the long photochromic ligand, 1,5-Bis(2-methyl-5-(4-pyridyl)-3-thienyl)cyclopentane (BPMTC), featuring two para-substituted pyridyl donors as the structural backbone, into crystalline MOP-based frameworks, was functionalization of **cuo**-MOP cages with dodecyl aliphatic chains leading to SBBs with a large hydrodynamic radius of approximately 3.4 nm, enabling subsequent interlinkage in an aliphatic solvent. The rationally designed DUT-210 series features cooperative photoswitchability as demonstrated by in situ PXRD and UV–vis experiments conducted upon light irradiation. Moreover, density functional theory (DFT) reveals differences in metal–ligand bond strength, providing access for tuning the switching kinetics in the isotropic topology. These findings open new possibilities for kinetic design in optically driven motors, actuators, or biomedical release system.

## Results and Discussion

### Synthesis and Characterization of MOPs

**Figure 2:**
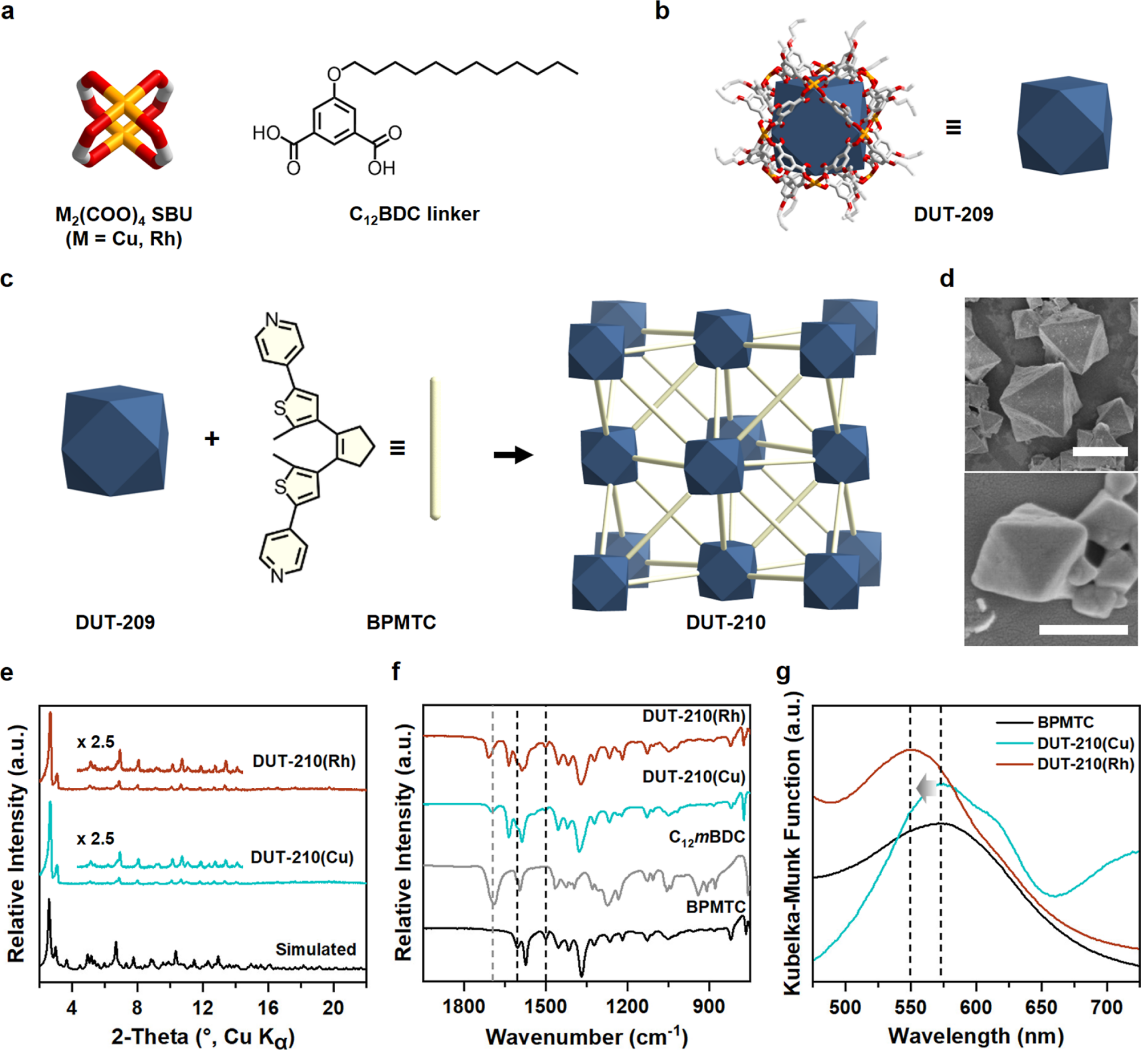
Self-assembly and characterizations of designed 3D photochromic MOFs based on MOPs and the BPMTC pillar. (a) Two structural building blocks of DUT-209; metal (Cu or Rh) paddlewheels and long alkyl chain-substituted C_12_*m*BDC linker. (b) cuo-MOP cage assembled from two components. Each vertex represents open metal site of nodes. (c) Reassembled DUT-210 series based on DUT-209 and the photochromic pillar, BPMTC. (d) SEM images of synthesized DUT-210(Cu) (top) and DUT-210(Rh) (bottom). The synthesized crystals represent an octahedral shape (scale bar: 10 μm and 500 nm, respectively). (e) Experimental PXRD data of DUT-210 series and simulated PXRD data. (f) FT-IR spectra of DUT-210 series and structural components; C_12_*m*BDC linker and BPMTC pillar. (g) DRS spectra which are irradiated on 365 nm of the DUT-210 series and BPMTC pillar.

To synthesize the novel 3D MOFs based on MOP cages and a linear bidentate ligand, we employed a two-step synthetic procedure. The MOPs were constructed using an alkoxylate derivative of the *m*BDC linker, 5-(dodecyloxy)-1,3-benzenedicarboxylic acid (C_12_*m*BDC), synthesized according to the established protocols^53^ (Figures S1–S3). We envisioned that increasing the hydrodynamic radius to about 3.3 nm^54^ by functionalizing the linkers of MOPs could help to form the 3D framework. The resulting MOP structures, namely, DUT-209, were synthesized by the combination of M_2_(COO)_4_ paddlewheel nodes (M = Cu and Rh) and C_12_*m*BDC by modifying the synthetic procedure (Figure 2a,b).^55,56^ The recrystallized DUT-209(Cu) crystals showed octahedral shapes with crystal sizes around 80 μm, while DUT-209(Rh) had uniform rhombohedral crystals with around 0.5 μm (Figure S4). Refined single-crystal X-ray diffraction (SCXRD) data reveal that DUT-209(Cu) is isostructural with MOP-18,^56^ though it exhibits slight differences in crystal packing due to variations in the recrystallization process. The PXRD patterns of the as-synthesized DUT-209(Cu) closely match the simulated PXRD patterns derived from SCXRD data, providing a better fit than the pattern from MOP-18 (Figure S5). The UV–visible absorbance spectra further confirmed the presence of dissolved MOP cages in the CHCl_3_ solvent (Figure S6). A weaker Cu^2+^ to ligand charge-transfer transition was observed at 679 nm,^57^ as expected for DUT-209(Cu), while for the Rh-based material, a characteristic absorption band of the Rh–Rh bond appeared at 626 nm.^50^ Also, the thermal stability of the DUT-209 series was confirmed through TGA and DTG analysis, showing the decomposition at 370 °C (Figure S7).

### Photochromic Ligand, BPMTC, and Analysis of Various Structural Forms

Prior to creating the extended 3D MOFs, the BPMTC ligand was synthesized via a five-step synthetic procedure (Figures S8–S13).^58−60^ To observe photoisomerization of the synthesized BPMTC, we used the output power of 365 and 550 nm LEDs operating at 100 and 110 mW, respectively. The free BPMTC ligand assumes an open-ring form with antiparallel configuration with respect to the transpositioning of the methyl groups attached to the two thiophene rings and hereby denoted as the *o*-form(*ap*), which transforms into a closed-ring form (hereafter denoted as the *c*-form) turning purple in color under the UV light. Under visible light, the *c*-form converted to the *o*-form(*ap*), decreasing the intensity of absorbance in UV–vis spectra as well as turning transparent from an intensive purple color solution (Figure S14). The DTE moieties of the ligand shows fast photoisomerization owing to a 6π-electron electrocyclic rearrangement resulting in the formation of a new C–C bond between the carbon atoms in the thiophene rings bearing the methyl groups.^61^ DFT calculations were performed to estimate the relative stabilities of the various structural forms of BPMTC (see Section S1.4 for computational details). Our results reveal that the *o*-form(*ap*) is around 9.7 kcal mol^–1^ more stable compared to the *c*-form. A substantially high thermal barrier for the mutual interconversion between the *o*-form(*ap*) and the *c*-form (91.1 kcal mol^–1^) is consistent with the fact that external stimuli such as light energy are required for this transformation (Figure S15). Additionally, BPMTC may exist in a different conformation, hereafter denoted as *o*-form(*p*), which is another open-ring form but with *cis*-positioning of the methyl(thiophene)rings (Figure S16). Based on our DFT calculations, the *o*-form(*p*) is 1.4 kcal mol^–1^ less stable compared to the *o*-form(*ap*) and, thus, present only in minor concentration at room temperature.

### Design and Synthesis of Photochromic MOFs

Various **cuo**-MOPs of 2 nm size have been reported consisting of 12 M_2_(COO)_4_ clusters (M = Cu, Rh, Ni, and Mo) and 24 R-*m*BDC (R = –H, –OH, –NO_2_, –SO_3_–, and –OC_12_H_25_) as organic linkers.^44−46^ Especially, **cuo**-MOPs have been utilized as SBBs forming 3D MOFs with **fcu-a** topology^42−45^ and as porous monomers establishing fascinating gel forms.^50−52^ The 12 axial positions of paddlewheel nodes in the MOP are coordinated with N-donor linear bidentate ligands of diverse lengths with 2.7–11.5 Å, as well as nonlinear bidentate ligands (Figure S17). However, establishing an extended framework with linear ligands exceeding 12 Å is extremely challenging because the formation of MOP packing in a molecular crystal tends to be energetically favorable, particularly as the length of the ligand increases, compared to coordination bond formation of metal to linear ligands, which induces the crystalline structure. In this regard, to construct extended 3D MOFs by assembling elongated ligands with **cuo**-MOPs, we considered key synthetic points: (i) the size of the discrete MOP cages is larger than 2 nm, or MOPs with long aliphatic chain-functionalized linkers are employed to increase the hydrodynamic radius of the cages^62^ and (ii) an aliphatic solvent is used to synthesize the MOF.

Based on these key points, novel 3D MOFs were successfully interconnected by photochromic BPMTC ligands and functionalized MOPs using mixed solvents with *N,N*′-dimethylacetamide (DMA) and 1-octanol (Figure 2c). The synthesized MOFs, namely, DUT-210(Cu) and DUT-210(Rh), exhibit characteristic colors. DUT-210(Cu) crystals are pale blue, while the DUT-210(Rh) crystals are red, as clearly observed in the optical microscopy (OM) images (Figure S18). Both MOFs exhibit octahedral-shaped crystals, as shown in the scanning electron microscopy (SEM) images (Figure 2d). Synchrotron SCXRD was applied to determine the crystal structures of DUT-210(Cu) and DUT-210(Rh), suggesting that the expected *Fmm* cubic space group and unit cell parameters could be determined. However, because of high degree of flexibility and disorder of alkoxy side chain of linker and BPMTC ligand, only the positions of MOPs could be unambiguously localized. The complete structural model was built based on the obtained structural information using the Materials Studio 5.0 software package. The PXRD data of the as-synthesized DUT-210(Cu) as well as DUT-210(Rh) show that the resulting structures are crystalline matching the simulated PXRD patterns (Figure 2e). The PXRD data of the as-synthesized DUT-210 were refined by the Pawley method (Figures S19 and S20). From the simulated structure, we observed that within a unit cell, 12 paddlewheel nodes of a MOP cage are connected with their neighboring MOPs via BPMTC ligands. ^1^H NMR analysis was performed to observe any ligand defect inside the MOF structure during the self-assembly process and to determine the ratio of C_12_*m*BDC and BPMTC in the acid-digested samples (Figure S21). The determined ratio of C_12_*m*BDC to BPMTC of both MOFs (DUT-210(Cu) = 4:1.06 and DUT-210(Rh) 4:1.1) agrees well with the theoretical ratio (4:1) found in the simulated structure. In addition, the incorporation of BPMTC and MOP units into the MOF structure was further confirmed through FT-IR spectra (Figures 2f and S22). The FT-IR spectra of DUT-210 exhibited characteristic vibrations, including *v*_s_(C=O) from the C_12_*m*BDC linker (gray line) and *v*_s_(C–N) and *v*_s_(C=C) from the BPMTC pillar (black lines). The coordination of BPMTC to the axial positions of the nodes causes a blue shift in the peak at around 50 nm compared to the free BPMTC molecule in the UV–vis diffuse reflectance spectroscopy (DRS) spectra (Figure 2g). Additionally, the incremental blue shift of band I from 626 to 530 nm proved the coordination with exterior and axial sites of Rh nodes toward N-donor ligands.^50^ The thermal stability of DUT-210(Rh) is comparable to that of DUT-209(Rh), showing the decomposition at 370 °C, while DUT-210(Cu) decomposes at 350 °C (Figure S23). These observations are consistent with theoretical calculations (see Section S1.4 for computational details), which estimate the ground-state bond dissociation enthalpy per formula unit to be −127.8 kcal mol^–1^ for DUT-210(Rh) and to be −111.3 kcal mol^–1^ for DUT-210(Cu) (Table S1 and Figure S24). The negative enthalpy values indicate that both MOFs are thermodynamically favorable. Notably, Rh-containing MOFs exhibit greater stability than their Cu counterparts, with a gain of 16.4 kcal mol^–1^. This increased stability for DUT-210(Rh) also suggests that these MOFs are likely to be more rigid compared with Cu-based MOFs.

The DUT-209 and DUT-210 series were activated using supercritical CO_2_ drying, followed by Ar flow treatment (Figure S25).^63^ The N_2_ physisorption of activated samples was conducted at 77 K, indicating that DUT-209 series are nonporous despite their crystallinity is maintained.^50^ Although the high porosity of DUT-210 was expected from geometrical calculations, DUT-210 systems showed low porosity for N_2_ at 77 K (Figure S26). The hysteresis loop, observed in the isotherm of DUT-210(Cu), closes at a relative pressure of *p*/*p*_0_ = 0.42, indicating the structure decomposition and formation of the mesopores. The isotherm of activated DUT-210(Rh) resembles supramolecular polymers showing loss of crystallinity.^50^ The bulky alkyl chain hinders diffusion of the gas through the MOP cavity and extrinsic pore in the framework. To understand the changes in the crystal structures upon desolvation, total scattering experiments were conducted on the solvated and desolvated DUT-210 samples. The comparison of the Fourier transformed pair distribution function (PDF) indicates no changes in the local structure of DUT-210(Rh), but minor changes in the local geometry of copper in DUT-210(Cu), especially for short distances up to 12 Å (Figure S27). In particular, two peaks at 2.0 and 2.4 Å can be attributed to Rh–O and Rh–Rh distances within one paddlewheel, respectively, showing no decomposition or distortion of the Rh paddlewheel sites for both solvated and desolvated DUT-210(Rh). However, the peak at 2.4 Å, which can be attributed to Cu···Cu distances within the paddlewheel of DUT-210(Cu), splits into two peaks at 2.4 and 2.8 Å after desolvation, showing that DUT-210(Cu) partially decomposed.

### Photochromic Behavior of DUT-210(Cu) and DUT-210(Rh)

**Figure 3:**
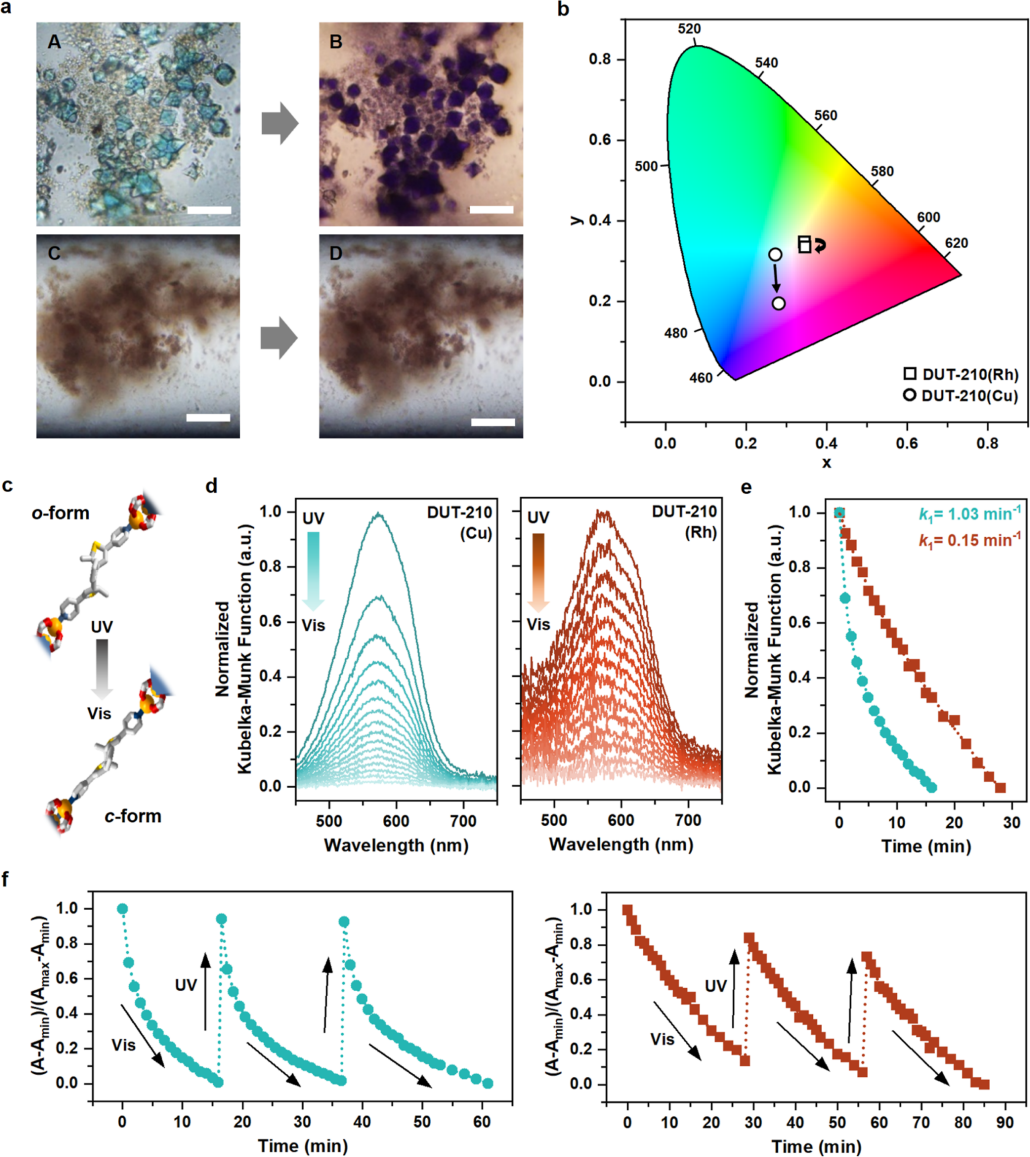
Photochromic behaviors of DUT-210. (a) Photochromic response of single crystals of DUT-210(Cu) (top) and DUT-210(Rh) (bottom) (scale bar: 50 μm). (b) CIE chromaticity diagram of the DUT-210 series before and after irradiation. (c) Schematic representation of the photoisomerization from the *c*-form to the *o*-form of BPMTC incorporated into the framework, illustrated by the decrease in peak intensity, as shown in Figure (d). (d) Normalized DRS spectra of the DUT-210 series. The samples were irradiated upon excitation with UV light (λ_ex_ = 365 nm) and subsequently exposed to visible light (λ_ex_ = 550 nm). (e) Photoisomerization kinetics and (f) normalized optical cycling studies of DUT-210 series.

To confirm the photochromic properties of both MOFs, we prepared single-crystal samples placed in quartz capillaries to prevent structural decomposition in the air during irradiation. A noticeable color change was observed in the crystals upon irradiation. As shown in Figure 3a, the pristine DUT-210(Cu) crystals have pale blue color. Immediately upon UV irradiation, the pale blue color turns to purple without any crystal shape changes. However, the purple color of the crystals gradually restored the original color under visible light, while the red crystals of DUT-210(Rh) similar to DUT-210(Cu) transitioned to dark red under the same conditions. The colors of both MOFs before and after irradiation were marked in the CIE diagram (Figure 3b). We investigated the photoisomerization kinetics of DUT-210 by in situ DRS spectra. The bulk samples were exposed to UV light, and then we monitored the attenuation of the colored photoisomers under visible light to calculate reverse rate constants, *k*_reverse_.^10^ As the peak intensity decreased, the *c*-form of BPMTC ligands incorporated into the framework was transformed to the *o*-form (Figure 3c,d). The initial stages of photoisomerization rate of MOFs have been the focus of recent investigations.^9−11^ When photochromic moieties are embedded as a structural backbone, the structural flexibility related to dimension and topology is an important factor to change the photoisomerization rate by controlling the photoswitching environment.^10,11^ We expected only minor variations in the measured rate constant for isostructural DUT-210(Cu) and DUT-210(Rh) frameworks because of in-plane isomerization of the DTE moiety.

However, surprisingly, the photoisomerization rates of DUT-210(Cu) and DUT-210(Rh) remarkably differed, namely, 1.03 and 0.15 min^–1^, respectively, demonstrating approximately 7-fold enhancement (Figure 3e). Furthermore, optical cycling was conducted to demonstrate the significant photoswitching fatigue resistance of both DUT-210 frameworks. Interestingly, during cycling, the photoisomerization of DUT-210(Cu) gradually slowed, while the photoisomerization behavior of DUT-210(Rh) remained consistently stable (Figures 3f and S28). Obviously, the coordination bonds between metal to the N-donor pillar differ significantly in dynamics. The significant difference in the computed electrostatic potential between Cu (137.32 au) and Rh (257.33 au) centers and relatively higher bond dissociation enthalpy (−127.8 vs −111.3 kcal/mol^–1^ for Rh and Cu, respectively) indicate stronger metal–ligand interactions for Rh-containing models which points to a more rigid framework (Figure S29 and Tables S2 and S3). Thus, we attribute the difference in the photoisomerization rate to the greater rigidity in the DUT-210(Rh), which restricts the structural adaptability of the framework during isomerization, resulting in faster conversion compared to the more flexible DUT-210(Cu). The flexibility in DUT-210(Cu), which is facilitated by weaker metal–ligand interaction, allows for greater structural rearrangement, thus modulating the photoswitching environment and reducing the isomerization rate.

### Light-Induced Structural Transformation of Photochromic MOFs

**Figure 4:**
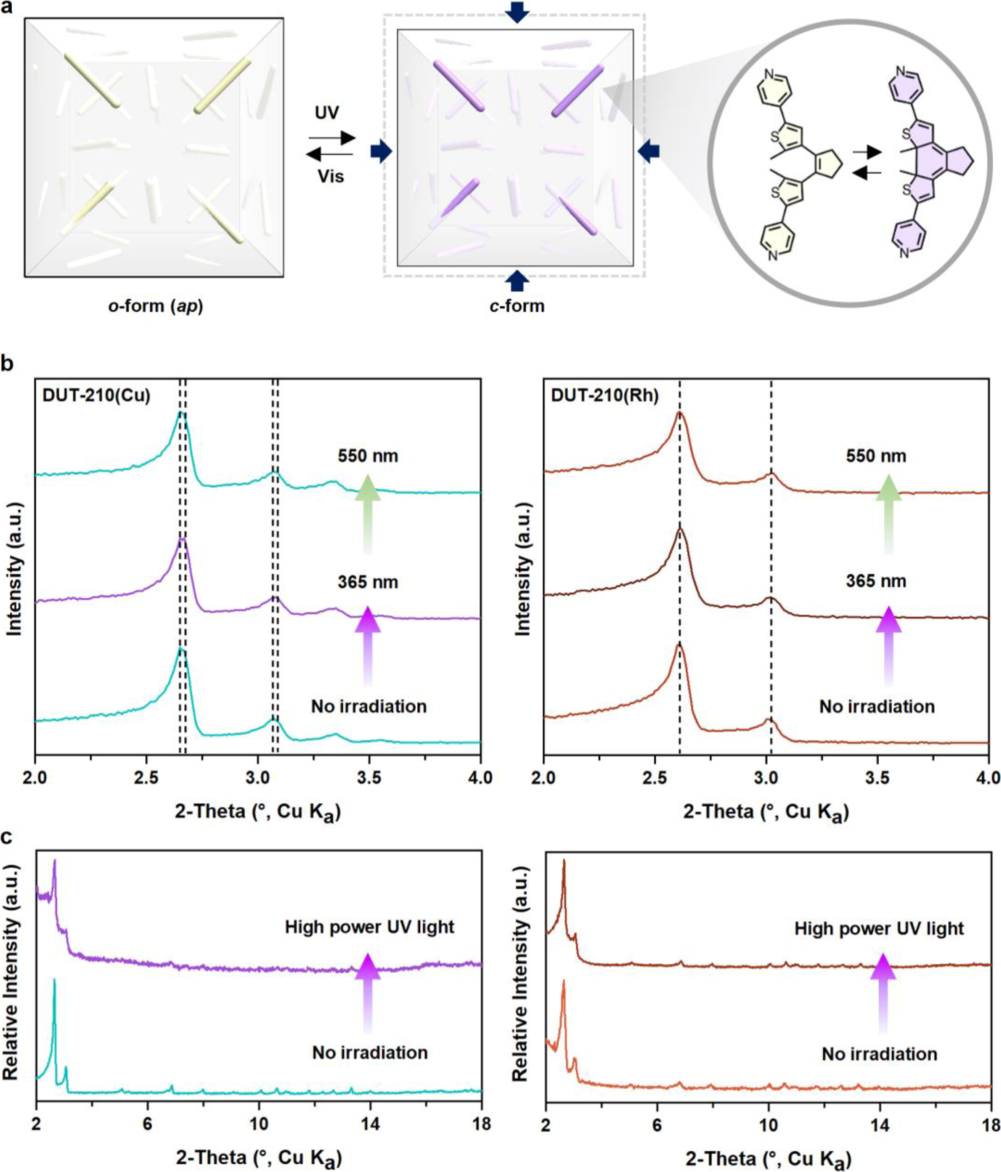
Photochromic structural transformation. (a) Schematic representation of cell shrinkage upon irradiation. The geometrical structures of the isomers in crystals. (b) In situ PXRD data of DUT-210 under the light irradiation. (c) Ex situ PXRD data of DUT-210 upon high power UV irradiation.

In contrast to the ordinary pillared layer MOFs incorporating BPMTC, which extended along the vertical direction,^27−30^ the BPMTC ligands in the DUT-210 series are arranged isotropically. We envisioned a distinguishable structural transformation, showing photoisomerization along all directions, not limited to one axis, although isomerization of DTE derivatives typically occurs within one plane with relatively minimal structural rearrangements in the framework (Figure 4a). To observe the structural transformation during the interconversion process under irradiation, in situ PXRD experiments were conducted using both MOFs. The crystals of DUT-210 series were filled in quartz capillaries with sufficiently small diameters of 0.3 mm to account for light penetration depth.^33^ The prepared capillaries were completely flame-sealed to prevent solvent evaporation, which could result in loss of crystallinity or guest-induced contraction upon desolvation.

The PXRD data were collected for two cycles under UV and visible irradiation to demonstrate the reversibility of complete photoisomerization. In the first cycle of DUT-210(Cu), the significant (111) peak in the PXRD data was slightly shifted by about 0.03°. To determine whether the length difference between the *o*-form(*ap*) and *c*-form of the BPMTC pillar affects this small peak shift, the cell parameters of the structure transformed to the *c*-form were recalculated, showing a cell shrinkage of about 0.46 Å. The small change of the cell parameter was reflected in the peak shift to a high angle in the PXRD data (Figure 4b). In contrast, for the DUT-210(Rh) sample, the peaks in the PXRD data were retained throughout the cycling process. The omnidirectional response of the DUT-210(Cu) structure is comparable to other pillared layer MOFs that incorporate the BPMTC ligand, which exhibits a unidirectional response. In comparison, the DAE-SURMOF structure contracts linearly in the [001] direction, resulting in a PXRD peak shift of 0.026° upon UV light irradiation.^30^ The structural change arises from a small distance change in the BPMTC ligand upon photoisomerization. Although this change is minimal and remains constant, its impact on the overall structure varies significantly depending on the spatial arrangement of the photochromic moiety. When the moiety is aligned in one direction, the resulting structural response is confined to that direction. However, when the moiety is distributed in multiple directions, the response propagates multiaxially. Thus, even a minor photoinduced change can generate diverse structural outcomes, depending on the alignment of the photochromic moiety within the framework. In parallel, when the photochromic moiety is aligned multiaxially within the framework, inherent structural flexibility and the types of photochromic moieties used are also crucial factors for enabling structural transformation upon light irradiation. For instance, DUT-163 is a notable example, embedding azobenzene as the photochromic moiety in all directions.^33^ The embedded azobenzene undergoes buckling, rather than the expected *E*–*Z* isomerization, to contract the structure upon irradiation. This occurs because the structural environment within the rigid framework suppresses the expected switching behavior of azobenzene molecules, limiting their movement and preventing the full rotation or bending required for isomerization. Interestingly, DUT-210 shares the same connectivity and topology as DUT-163. The key difference lies in the type of photochromic moiety, such as the azobenzene-based linker or the DTE-based N-donor ligand, connecting the MOPs. The internal arrangement of these moieties generates distinct photoswitching behaviors.

Interestingly, in a second cycle of the DUT-210 (Cu) sample, an unexpected change was observed in the PXRD data after irradiation with visible light for over 24 h. New peaks appeared, and the overall intensity decreased. Ultimately, the X-ray diffraction pattern transformed entirely, no longer matching that of the as-synthesized structure in its initial state (Figure S30). This unexpected outcome was confirmed through ex situ experiments using high-power UV light for 30 min. Similar to the in situ PXRD results, DUT-210(Cu) lost its crystallinity, whereas DUT-210(Rh) retained its crystallinity (Figure 4c). The structural stability of DUT-210(Rh), enhanced by the strong bond strength between Rh and the N-donor ligand, enables it to withstand intense UV light, demonstrating its potential for use in high-power UV sensing applications. In contrast, the fragile DUT-210(Cu) is unsuitable for such conditions. The photoisomerization of the DTE moiety in these MOFs exhibits notable thermal stability, which distinguishes them from other photochromic moieties such as azobenzene and spiropyran. Ground- and excited-state DFT calculations on truncated models of DUT-210 show that the reaction energy for the conversion from *o*- to *c*-form is excessively high, and this process is less likely to be thermally activated. However, in the electronic excited state, the system gains enough energy from photoactivation to drive the conversion (Tables S4 and S5). Thus, under strong UV light for short durations or weak UV light for prolonged periods, the structural transformation of DUT-210(Cu) is driven solely by light exposure rather than thermal activation. Excessive light energy induces a complete and irreversible structural change. All photochromic MOFs incorporating DTE derivatives as structural backbones have been reported to show photoisomerization between the *o*-form(*ap*) and *c*-form. DTE derivatives fixed in the framework hampered adoption of the *o*-form(*p*) conformations, unlike in molecular systems where the two conformations are freely reversible. In contrast, structural reorganization allowing various conformations in discrete cages, as demonstrated by Clever’s group in 2016,^64^ show that these cages can exist without external connectivity, unlike extended frameworks. Interestingly, discrete cage-based DUT-210(Cu) exhibits different photoisomerization behaviors due to its structural flexibility, in contrast to the rigid DUT-210(Rh), despite their isostructural nature.

## Conclusions

In summary, we successfully synthesized 3D photochromic MOFs, denoted as DUT-210(Cu) and DUT-210(Rh), by assembling MOPs and photochromic ligands. To the best of our knowledge, a crystalline RhMOP-based 3D MOF-embedded photochromic ligand is reported for the first time. The introduction of MOPs as SBBs leads to the development of new types of 3D photochromic and dynamic MOFs, unifying photochromic behavior and switching between the *o*-form and *c*-form due to the presence of M-N bonding. Our results provide insights into controlling the kinetics of photoisomerization in different metal-incorporated MOP-based MOFs and highlight the potential of MOPs as versatile scaffolds for dynamic, photoresponsive materials. Furthermore, the structural rigidity of MOP-based MOFs, which varies based on the bond strength within the framework, enables the development of UV light sensors that can operate across a wide range of intensities. This study opens avenues for developing photochromic MOFs with customizable switching properties and suggests possible applications in energy transfer and UV sensing by immobilizing various photosensitizers within discrete MOP cages. Future research could further explore the design of MOP-based MOFs with diverse photoswitchable behaviors, enabling advances in stimuli-responsive material applications.

## Supporting Information Available

Materials and methods, synthetic procedure, and characterization data; spectroscopic data (^1^H NMR, IR, and UV–vis); and in situ PXRD, DRS, and SCXRD refinement details (PDF)
All isotherm data were generated in *.aif format^65^ (ZIP)

## Abbreviations

MOPs: metal–organic polyhedra
MOF: metal–organic framework
DTE: diarylethene
SBBs: supramolecular building blocks
BDC: benzenedicarboxylic acid
BPMTC: 1,5-Bis(2-methyl-5-(4-pyridyl)-3-thienyl)cyclopentane
C_12_*m*BDC: 5-(dodecyloxy)-1,3-benzenedicarboxylic acid
DUT: Dresden University of Technology
**cuo**-MOPs: cuboctahedral MOPs
DMA: *N,N*′-dimethylacetamide
PDF: pair distribution function
PXRD: powder X-ray diffraction
SCXRD: single-crystal X-ray diffraction
OM: optical microscopy
SEM: scanning electron microscopy
DRS: diffuse reflectance spectroscopy
