# Supplementary material for: Engineering Photoswitching Dynamics in 3D Photochromic Metal–Organic Frameworks through a Metal–Organic Polyhedron Design
Source: J Am Chem Soc. 2025 Feb 25;147(10):8568–77. doi: 10.1021/jacs.4c17203 (PMC11912325; doi:10.1021/jacs.4c17203)
Supplement: Supplementary file 1 — ja4c17203_si_001.pdf [file ja4c17203_si_001.pdf]

Supporting Information

Engineering Photoswitching Dynamics in 3D Photochromic Metal-Organic Frameworks through Metal-Organic Polyhedron Design

Eunji Jin[*,†], Volodymyr Bon[†], Shubhajit Das[‡], A. D. Dinga Wonanke[‡], Martin Etter[§], Martin A. Karlsen[§], Ankita De[†], Nadine Bönisch[†], Thomas Heine[‡,‖,⊥], Stefan Kaskel[*,†]

[†]Chair of Inorganic Chemistry I, Faculty of Chemistry and Food chemistry, Technische Universität Dresden, Bergstraße 66, 01069 Dresden, Germany

[‡]Chair of Theoretical Chemistry, Faculty of Chemistry and Food chemistry, Technische Universität Dresden, Bergstraße 66c, 01069 Dresden, Germany

[§]P02.1 Beamline, PETRA III, Deutsches Elektronen-Synchrotron DESY, Notkestraße 85, 22607 Hamburg, Germany

[‖]Institute of Resource Ecology, Helmholtz Zentrum Dresden-Rossendorf, Permoserstraße 15, 04318 Leipzig, Germany

[⊥]Department of Chemistry, Yonsei University, Seodaemun-gu, Seoul 120–749, Republic of Korea

Corresponding author: eunji.jin@tu-dresden.de and stefan.kaskel@tu-dresden.de

Contents

1. **Materials and Methods**

2. **Synthesis of 5-(dodecyloxy)-1,3-benzenedicarboxylic acid (C_{12}*m*BDC) linker**

3. **Characterization of MOPs, DUT-209(Cu) and DUT-209(Rh)**

4. **Synthesis of 1,5-Bis[2-methyl-5-(4-pyridyl)-3-thienyl]cyclopentane (BPMTC) pillar**

5. **Supramolecular materials via assembly of MOPs and *N*-donor bidentate ligands**

6. **Characterization of MOFs, DUT-210(Cu) and DUT-210(Rh)**

7. **Photochromic properties of DUT-210**

8. **References**

1. Materials and Methods

1.1 Materials

All the chemicals were purchased from commercial sources. Rhodium(II) acetate, dimer, (98+%, TCI), 1-octhanol (99%, Fisher Scientific), *N*,*N*-Dimethylformamide (99.50%, Fisher Scientific), *N*,*N*-Dimethylacetamide (99%, Sigma-Aldrich), Copper(II) Acetate Monohydrate (>95.0%, TCI), Dimethyl 5-hydroxyisophthalate (98%, Alfa Aesar), 2-Methylthiophene (98%, Sigma-Aldrich), *N*-chlorosuccinimide (98%, Sigma-Aldrich), Glutaryl dichloride (97%, Sigma-Aldrich), Aluminum chloride anhydrous (98.50%, Merck), Carbon disulfide (99.90%, Alfa Aesar), Titanium(III) chloride tetrahydrofuran complex (97%, Thermo Scientific), Zinc (dust, <10 µm, ≥98%, Sigma-Aldrich), Magnesium sulfate anhydrous (99%, Grüssing GmbH), Tetrahydrofuran anhydrous (>99.0+%, TCI), n-Butyllithium (2.5M in hexanes, Sigma-Aldrich), Tributyl Borate (99.0+%, TCI), Tetrakis(triphenylphosphin)palladium(0) (99.0%, Sigma-Aldrich), 4-bromopyridine hydrochloride (99%, alfa Aesar), Hydrochloric acid (ACS Reagent, ≥37%, Honeywell Fluka), 1-bromododecane (>98.0%, TCI), Acetic acid (ROTIPURAN® 100 %, p.a., Carl Roth GmbH), Celite®545 (Particle size 0.02–0.1 mm, Merck), Potassium carbonate anhydrous (≥99.5%, Fisher Scientific), Sodium carbonate anhydrous (99.5%, Grüssing GmbH), Sodium hydroxide (≥97.0%, VWR Chemicals), Potassium hydroxide (86%, Analytic Reagent, Fisher scientific), Ethylene glycol (≥99.5%, Carl Roth GmbH), Methanol (≥99.8%, VWR Chemicals), Tetrahydrofuran (≥99.0%, Sigma Aldrich), Diethyl ether (>95%, Fisher Scientific), Chloroform (>99%, Fisher Scientific), Chloroform-d (99.8%, Deutero GmbH), DMSO-d_6 (99.8%, Deutero GmbH). The 5-(dodecyloxy)-1,3-benzenedicarboxylic acid (C_{12}*m*BDC) linker and 1,5-Bis[2-methyl-5-(4-pyridyl)-3-thienyl]cyclopentane (BPMTC) pillar were synthesized, followed by modified recipes as shown in Section S2 and S4.

1.2 Methods

Synchrotron single crystal X-ray diffraction (SCXRD). Single crystals of DUT-209(Cu) were prepared in a quartz glass capillary (*d* = 0.3 mm) with a small amount of solvent. The data set was collected at the BL14.2 beamline of the BESSY II synchrotron, operated by Helmholtz-Zentrum Berlin für Materialien and Energie.[1] Four images from different crystal orientations were collected in order to determine the crystal symmetry and scan angle range using the iMosflm program.[1,2] The φ scan with an oscillation step of $\Delta\varphi$ = 0.1° was used for the collection of 1800 frames, which were processed automatically using the XDSAPP 2.0 software.[1,3] The crystal structures were solved by direct methods and refined by full matrix least squares on F^2 using the SHELX-2018/3 program package.[1,4] All non-hydrogen atoms were refined in an anisotropic approximation. Distance and thermal parameter restraints were used to model the disordered alkoxy-chains. Hydrogen atoms were refined in geometrically calculated positions using a “riding model” with $U_{iso}(H) = 1.2U_{iso}(C)$.

CCDC 2401155 contains the Supporting crystallographic data for DUT-209(Cu) These data can be obtained free of charge from the Cambridge Crystallographic Data Centre via www.ccdc.cam.ac.uk/data_request/cif.

Crystallographic Data for DUT-209(Cu): $C_{284}H_{208}Cu_{24}N_4O_{144}$, M = 7505.49 g mol^{-1}, tetragonal, $I4_1/a$ (no. 88), a = 35.100(5) Å, c = 67.520(14) Å, V = 83185(29) Å^3, Z = 4, λ = 0.82656 Å, T = 293 K, θ_{max} = 27.665°, reflections/parameter 30766/909, R_{int} = 0.0603, for I/σ(I) >2: R_1 = 0.1332, wR_2 = 0.3991, S = 1.285; for all data: R_1 = 0.1787, wR_2 = 0.4527, S = 1.291; largest diff. peak 0.973 e Å^{-3} and hole -0.593 e Å^{-3}.

Powder X-ray diffraction (PXRD). PXRD was measured on a STOE STADI P diffractometer which can be built-up in transmission geometry using a rotating flatbed sample holder and operated at 40 kV and 30 mA with Cu-$K_{\alpha 1}$ radiation (λ=1.5406 Å) and 1D detector (Mythen, Dectris) at room temperature. The exposure time was 120 seconds per step and 0.015° steps.

Nuclear magnetic resonance spectroscopy (NMR). ^{1}H NMR and ^{13}C NMR data were obtained using a Bruker AVANCE 300 MHz spectrometer.

Scanning electron microscopy (SEM). Prior to measurement, Au was used for sputtering for the sample preparation. SEM measurement was conducted using 2.0 kV acceleration voltage and a working distance of approximately 8.0 mm on a SU8020 from Hitachi.

Thermogravimetric analysis (TGA). TGA was performed in a temperature range from 40 °C to 800 °C with a heating rate of 5 °C min^{-1} under Ar flow using a Netzsch STA 449 F5.

Fourier-transform infrared (FT-IR) spectroscopy. FT-IR spectra were recorded in ATR mode using a BIORAD Excalibur FTS3000 (Varian Inc.) infrared spectrometer.

High-resolution mass spectrometry (HRMS). HRMS was obtained using Bruker impact II ultra-high resolution QTOF quadrupole time-of-flight analyzer.

Liquid UV-Vis spectroscopy. Liquid UV-Visible absorbance spectra were obtained on a Shimadzu UV-1650PC spectrophotometer equipped with a mercury lamp.

Supercritical CO_2 drying process. Prior to the supercritical drying process, the reaction product was washed with mother liquid and exchanged with acetone which is miscible with liquid CO_2. The acetone was replaced and the samples were placed in a Jumbo Critical Point Dryer 13200J AB (SPI Supplies). The acetone was exchanged with liquid carbon dioxide (purity: 99.995 %) at 17 °C for 3 days. After that the temperature was increased to 40 °C and the supercritical CO_2 was slowly released over 1 h. After that the autoclave was flushed with Ar gas for 30 minutes to replace to CO_2 in the pores. The dried samples were transferred to an Ar-filled glove box.

Gas physisorption. Prior to all physisorption measurements, the samples were activated using supercritical CO_2 and flushing Ar and evacuated at low temperature. N_2 physisorption isotherms were measured up to 1 bar on a BELSORP-max II instrument by MICROTRAC MRB.

Pair distribution function (PDF) analysis. Total scattering experiments were conducted at beamline P02.1, PETRA III, DESY. An X-ray wavelength of 0.207361 Å was used. The signal of the scattered X-rays was collected using a Varex XRD 4343CT (150 × 150 μm^2 pixel size, 2880 × 2880 pixel area, CsI scintillator directly deposited on amorphous Si photodiodes) detector. The detector was placed in 'corner configuration', i.e., with the beam center oriented at the lower right corner of the detector, looking downstream from the sample position towards the detector. For calibration of the experimental geometry, a special glass (soda lime glass) capillary with an internal diameter of 0.8 mm containing LaB6 SRM 660c NIST standard powder was used. The sample-to-detector distance (SDD) was calibrated to be 300.613 mm using the pyFAI software.[5]The sample powders were prepared in borosilicate capillaries with internals diameters of 0.5 and 0.7 mm. To measure the background contribution to the total scattering signal, empty capillaries were measured. The samples and the empty capillaries for background measurements were exposed to X-rays for 25 minutes. The data were azimuthally integrated using the pyFAI software. For each individual sample, the corresponding background signal was scaled to account for X-ray beam intensity fluctuations and subtracted from the total signal for the samples, prior to further data reduction, including final Fourier transformation to obtain the reduced atomic pair distribution function (PDF), using the PDFgetX3 algorithm[6] through the xPDFsuite software.[7]

Light-emitting diodes for irradiation. For irradiation studies, LEDs from Mountain Photonics (Prizmatix FC5-LED Multi channel LED light source were used. The detailed specifications of the LEDs include an output power of 100 mW at 365 nm and 110 mW at 550 nm (measured with a POF: 1000 μm core diameter, NA = 0.5, L = 1 m). The light power density of the LEDs without a POF was measured using an optical power meter (MC-PM100C from Beijing MerryChange Technology Co., Ltd), indicating optical power density without the POF was 64 mW/cm^2 for 365 nm and 52 mW/cm^2 for 550 nm wavelengths at a distance of 0.8 cm.

Light induced *in situ*-UV-Vis spectroscopy. Liquid UV-Vis spectra of BPMTC solution which was prepared in $CHCl_3$ were obtained on a Shimadzu UV-1650PC spectrophotometer equipped with a mercury lamp. Diffuse reflectance UV-Vis spectra were recorded using a VARIAN CARY 4000. DUT-210 samples were washed three times over two days with 12 mL of fresh mother liquid, DMF/1-octhanol (1:1, v/v). The washed samples were then filtered. A total of 9 mg of the DUT-210 series was homogeneously mixed with 160 mg of BaSO4 using a mortal. The prepared samples were placed in a sample well with a diameter of 0.6 cm and a depth of 0.1 cm (surface area: 0.3 cm^2). To prevent solvent evaporation, a HARRICK Praying

Mantis reaction chamber was equipped with a dome containing UV-Vis transparent glass observation windows. The attenuated spectra were recorded over a wavelength range of 400–800 nm. The LED was installed perpendicular to the windows at a distance of 0.8 cm.

Light induced *ex situ*-optical microscopy. To observe photochromic behavior of DUT-210 series under the optical microscopy during irradiation, the samples were prepared by followed. As-synthesized samples were washed with 12 mL of fresh mother liquid, DMA/1-octhanol (1:1, v/v) for three times for two days. The washed MOF crystals placed in the quartz capillary (0.3 mm of outside diameter and 0.01 mm of wall thickness) and the capillaries were sealed to prevent the structural decomposition in the air during the irradiation. The color of crystals was observed before and after irradiation under the optical microscopy.

Light induced *in situ*-PXRD. The synthesized samples were washed with 12 mL of fresh mother liquid, DMA/1-octhanol (1:1, v/v) for three times for two days. The washed samples were filled in quartz capillary (0.3 mm of outside diameter and 0.01 mm of wall thickness). The prepared capillary was flame sealed to prevent solvent evaporation. *In situ*-PXRD data were collected from a STOE STADI P diffractometer equipped with Cu-$K_{\alpha 1}$ radiation (λ= 1.5406 Å) and 2D detector (Mythen, Dectris) at room temperature. The measurements were conducted in a transmission geometry using a capillary spinner and exposure time of 200 seconds per step and 3° 2θ steps. The LED was installed perpendicular to quartz capillary with distance of 0.5 cm.

Light induced *ex situ*-PXRD. The synthesized samples were irradiated with strong UV light for 30 minutes. The reacted samples were then measured using the same device as for the *in situ*-PXRD data.

1.3 Synthesis of MOPs and MOFs

DUT-209(Cu). DUT-209(Cu) was synthesized by modified recipes.[8] C_{12}*m*BDC (0.147 g, 0.42 mmol) and $Cu(OAc)_2 \cdot H_2O$ (0.082 g, 0.41 mmol) in DMF solvent (10 mL) were dissolved in a Pyrex tube (12 mL). The mixed solution was sonicated and reacted at room temperature for 48 hours. Blue needle shape crystals were formed. All of the crystals were collected and DMF solvent was removed after centrifuging. The collected crystals were dissolved in fresh acetone (10 mL) for recrystallization at room temperature for 24 hours. After recrystallization, light blue colored crystals of octahedral shape were formed.

DUT-209(Rh). DUT-209(Rh) was synthesized by a modified synthetic process.[9,10] C_{12}*m*BDC (0.174 g, 0.5 mmol) and $Rh_2(OAc)_4$ (0.08 g, 0.2 mmol) in the presence of Na_2CO_3 (0.052 g, 0.5 mmol) were dissolved in 10 mL of *N*,*N*-dimethylacetamide (DMA) and 16 µL of MeOH using a Pyrex tube (12 mL). The mixed solution was sonicated and reacted at 100 °C for 24 hours in a pre-heated oven. The resulting green solution

was filtered and the supernatant treated with MeOH to recrystallize the DUT-209 crystals for 24 hours. After recrystallization, dark green colored crystals of octahedral shape were formed.

DUT-210(Cu). The recrystallized DUT-209(Cu) was centrifuged and the acetone was removed in the vial. The collected DUT-209(Cu) crystals were dissolved in DMA/Octanol (1:1, v/v), 12 mL (Solution A). The Solution A was divided into 6 Pyrex tubes and BPMTC (23 mg, 0.05 mmol) was added in each tube. The mixed solution was sonicated and reacted at 100 °C for 24 hours in a pre-heated oven. After cooling, octahedron shaped-pale blue crystals were formed.

DUT-210(Rh). The synthesis of DUT-210(Rh) was followed by the same procedure with DUT-210(Cu). Red colored crystals were formed in the vial.

1.4 Computational studies

Molecular geometry optimization. The molecular geometries of the *o*- and *c*- forms of the BPTMC ligands were optimized using the PBE0[11,12] exchange-correlation functional augmented by the addition of the D3(BJ) dispersion correction[13,14] as implemented in the Gaussian 16 software.[15] For optimization, the def2-SVP basis set was used, while further single-point calculations were performed with a larger def2-TZVP basis set to refine the electronic energies.[16]

Bond dissociation enthalpy. To compute the bond dissociation energies, we performed single-point energy calculations on the as-synthesized crystal structure using the GFN-xTB method (Geometry, Frequency, and Non-covalent Tight Binding).[17] This is because the GFN-xTB is a good approximation to first-principles density functional theory (DFT) that utilizes precomputed parameters, including third- and fourth-order integrals, for elements up to Z = 87 in the periodic table. This method allowed us to approximate the dissociation enthalpies of the MOPs at reasonable computational costs for such a large system. For each MOP, two different deconstruction approaches were employed to compute the bond dissociation enthalpy. In the first approach, the MOPs were broken down into their unique metal clusters and organic ligands. In this formalism, the bond dissociation enthalpy reflects the strength of the bond formed between the ligands and the metal clusters, which corresponds to the energy required to assemble the primary units. In the second approach, the MOPs were deconstructed into their unique metal and organic secondary building units. Here, the bond dissociation enthalpy represents the energy needed to assemble the SBUs into their underlying topologies within the overall structure. The bond dissociation enthalpy was computed using Equation S1 and the enthalpies reported in Table S1 correspond to the enthalpy of one formular unit.

$$\Delta H_{dis} = E_{MOP} - (xE_{metal} + yE_{pillar} + zE_{linker}) \quad \text{Equation S1}$$

Where

- ΔH_{dis} is the dissociation enthalpy of the MOP
- E_{MOP}, E_{metal}, E_{pillar}, and E_{linker} represent the electronic energies of the MOP, metal cluster/metal SBUs, BPMTC pillars and C_{12}*m*BDC ligand/SBU respectively.
- x, y and z represent their stochiometric ratios in the MOP, which corresponds to 48, 24 and 96 respectively. This corresponds to the number of these entities found in the unit cell.

The enthalpy for one formular unit is obtained by diving the total enthalpy by three since each MOP is made from 3 unique building units. These building units are represented in Fig. S23.

Truncated model. Due to the computational limitations associated with the large size of MOFs with more than 6000 atoms in the unit cell, we truncated the structure to a more manageable model containing key fragments that are essential for understanding the photodynamic ring closure. Specifically, we created four truncated fragments representing both the open and closed configurations of the Cu- and Rh-containing variants of DUT-210. Each truncated fragment consists of two paddlewheel SBUs separated by a BPMTC pillar. To ensure system neutrality, hydrogen atoms were added at all C_{12}*m*BDC point of extension. The truncated models for the open and closed systems are illustrated in Fig. S28. For each truncated model, we performed a geometry optimization in the ground electronic state using the PBE-D3/TZP level of theory, incorporating ZORA spin-orbit coupling to account for relativistic effects due to the heavy metals. Subsequently, we performed single-point time-dependent density functional theory (TDDFT) calculations for the excited states, using the same level of theory as the ground state calculations.[18] However, unlike the ground-state calculations, we could not include relativistic effects in the excited-state computations, as spin-orbit coupling is not permitted for excited-state calculations at this level of theory in the Amsterdam Modeling Suite package. The default Davidson method was used to solve the time-dependent eigenvalue equation, as it provides a reasonable balance between computational cost and accuracy.[19] We calculated the 30 lowest excited states for both singlet-singlet and singlet-triplet transitions. All GFN-xTB, ground, and excited state DFT calculations were performed using the Amsterdam Modelling Suite package (version 2024). Unless otherwise stated, default settings were used for all calculations.[20]

2. Synthesis of 5-(dodecyloxy)-1,3-benzenedicarboxylic acid (C_{12}*m*BDC) linker

C_{12}*m*BDC linker functionalized with the long alkyl chain was synthesized by modified recipes.[21] The synthetic process of two steps is followed by Fig. S1.

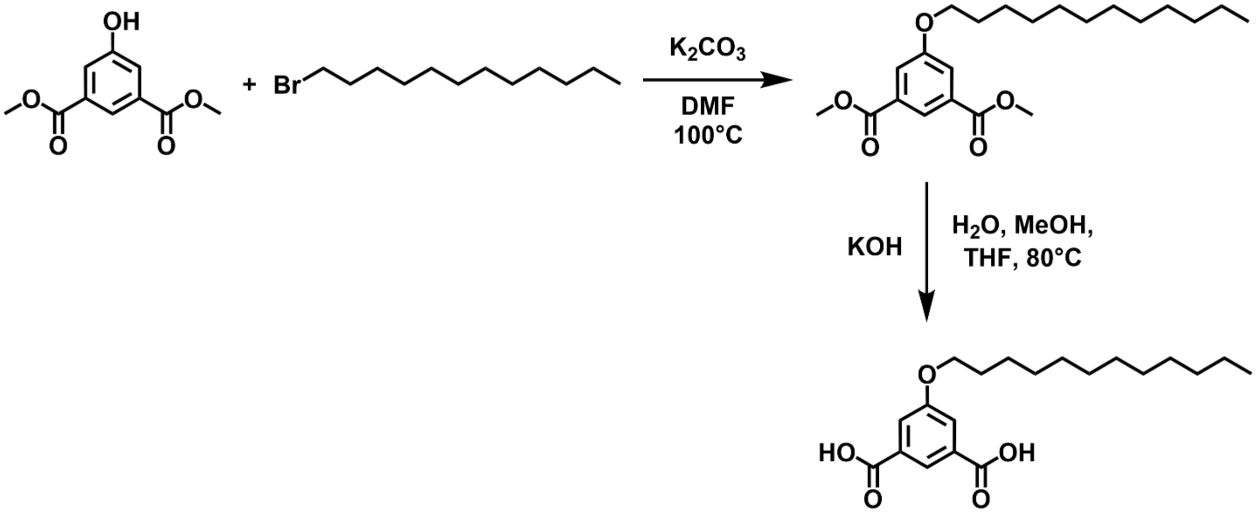

Supporting Figure 1. Synthetic process of C_{12}*m*BDC linker.

2.1 Synthesis of 5-(dodecyloxy)isophthalic acid methyl ester. 2 g of dimethyl 5-hydroxyisophthalate (9.5 mmol) and 3.94 g of K_2CO_3 (28.5 mmol) in 50 mL of DMF and then, 2.55 mL of 1-bromododecane (10.6 mmol) were added into the mixture. The mixture was heated at 100 °C for 24 hours. After reaction, DMF was removed using a rotary evaporator and H_2O was added to the residue to obtain a white dispersion. The precipitated product was filtered out and washed with H_2O. The resulting solid was dried at 85 °C for 24 hours. ^1H NMR (300 MHz, $CDCl_3$): δ = 8.19 (t, *J* = 1.4 Hz, 1H), 7.67 (d, *J* = 1.4 Hz, 2H), 3.97 (t, *J* = 6.5 Hz, 2H), 3.88 (s, 6H), 1.88–1.68 (m, 2H), 1.56–1.06 (m, 20H), 0.83 (t, *J* = 6.7 Hz, 3H) ppm.

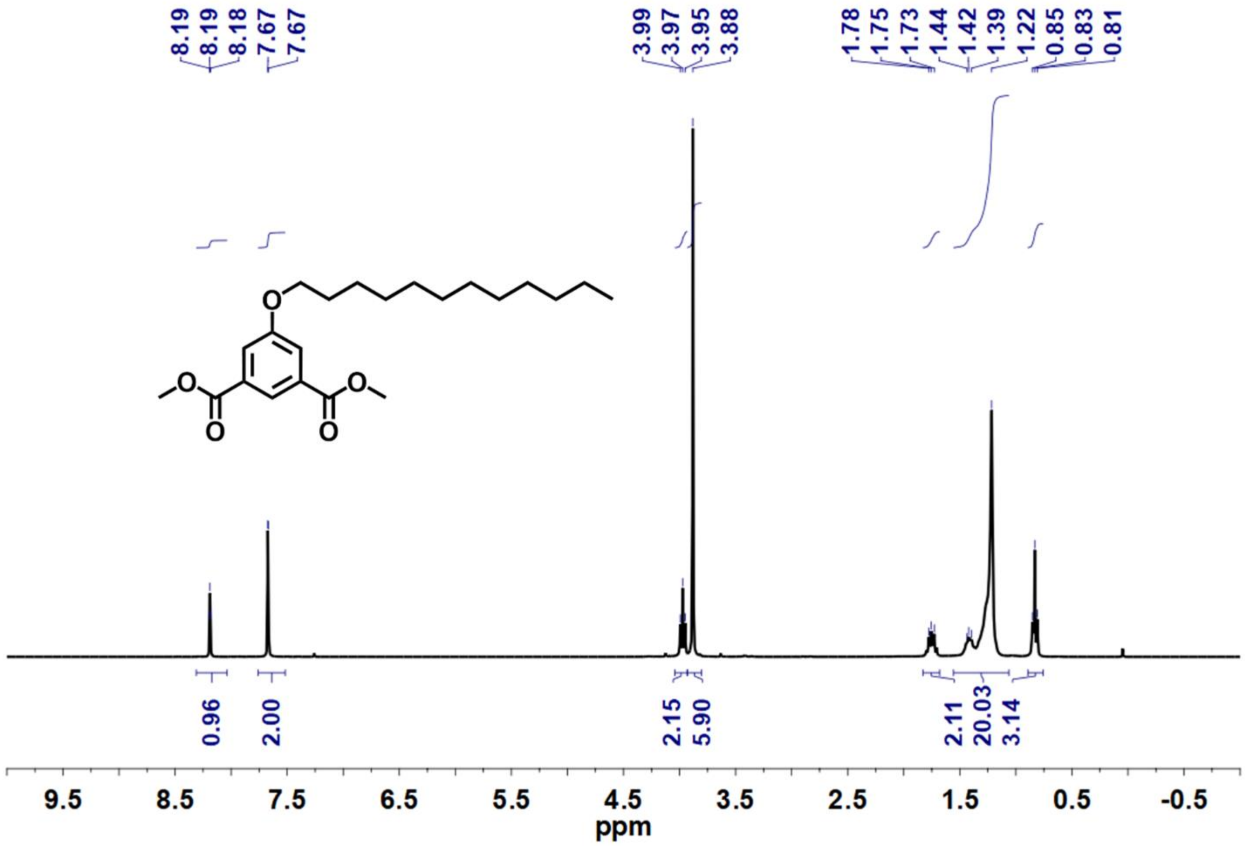

Supporting Figure 2. ^1H NMR spectrum of 5-(dodecyloxy)isophthalic acid methyl ester.

2.2 Synthesis of C$_{12}$*m*BDC. The dried sample was dissolved into 40 mL of THF, 40 mL MeOH, and 30 mL of 2 M KOH aqueous solution. The mixture was heated at 85 °C for 24 hours under reflux. Afterwards, MeOH and THF solvents were removed under reduced pressure using a rotary evaporator. The residue solution was acidified with 3M HCl forming a white precipitate. And then the white product was filtrated and washed several times with H_2O, and finally dried at 85 °C in the oven. (3 g, 97 %). ^{1}H NMR (300 MHz, DMSO-d_6): δ = 8.06 (t, *J* = 1.3 Hz, 1H), 7.61 (d, *J* = 1.3 Hz, 2H), 4.03 (t, *J* = 6.4 Hz, 2H), 1.83–1.58 (m, 2H), 1.53–1.03 (m, 18H), 0.82 (t, *J* = 6.7 Hz, 3H) ppm.

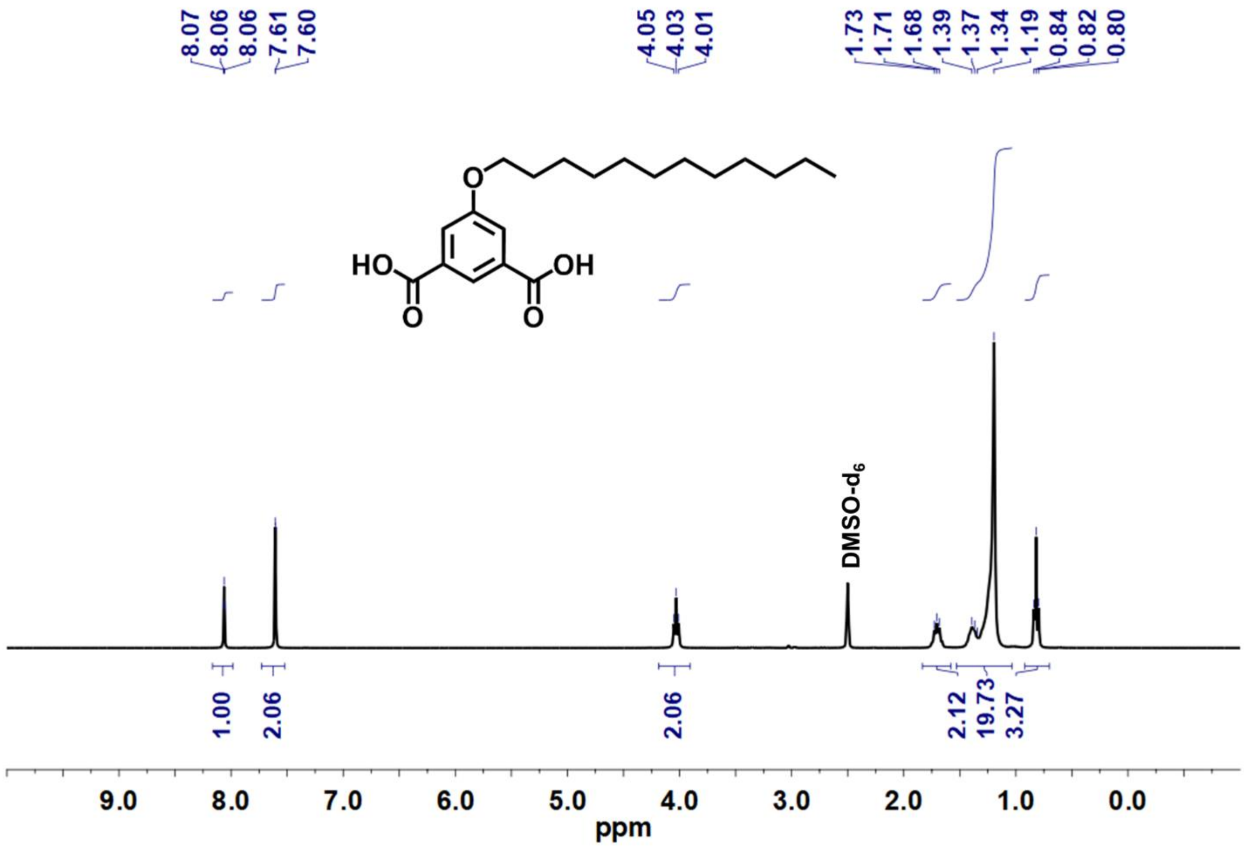

Supporting Figure 3. ^{1}H NMR spectrum of C$_{12}$*m*BDC linker.

3. Characterization of MOPs, DUT-209(Cu) and DUT-209(Rh)

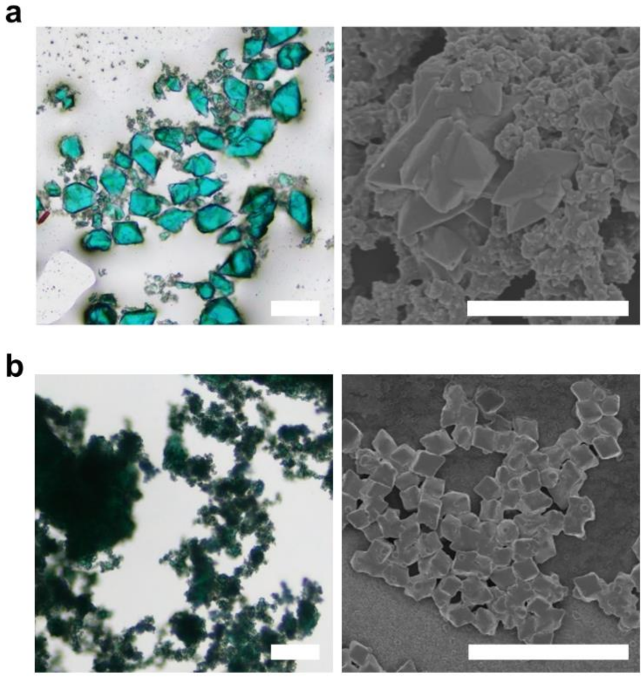

Supporting Figure 4. Optical microscopic and SEM images of DUT-209. a, OM and SEM images of DUT-209(Cu). The light blue colored crystals of uniform octahedral shape were obtained after recrystallization in acetone (Scale bar: 100 and 3 μm, respectively). **b,** OM and SEM images of DUT-209(Rh). The dark green colored crystals show uniform rhombohedral shapes after recrystallization in methanol (Scale bar: 100 and 30 μm, respectively).

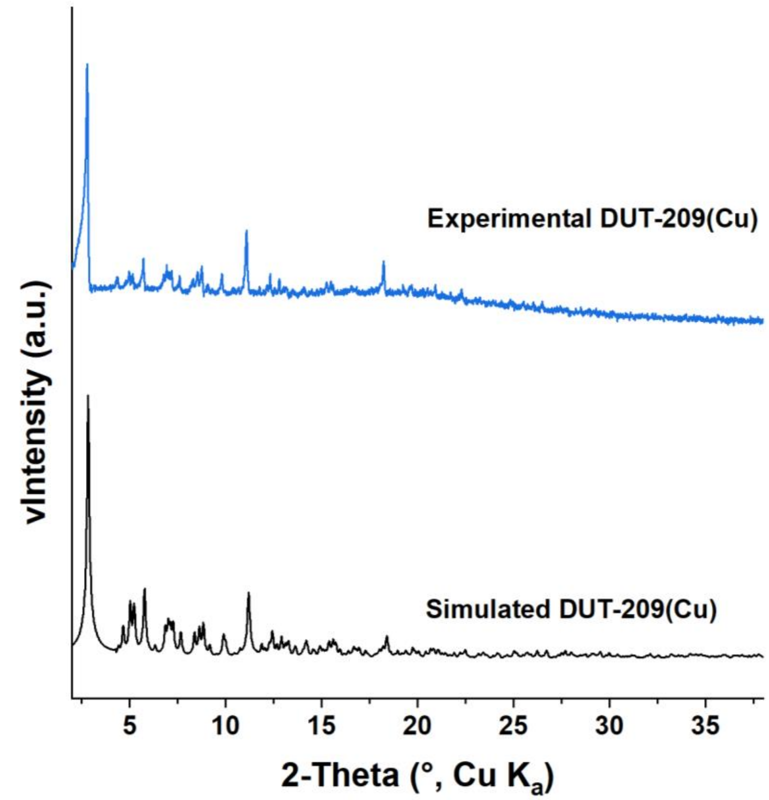

Supporting Figure 5. Powder X-ray diffraction data of DUT-209(Cu). Experimental PXRD patterns of DUT-209(Cu) was matched with the simulated one.

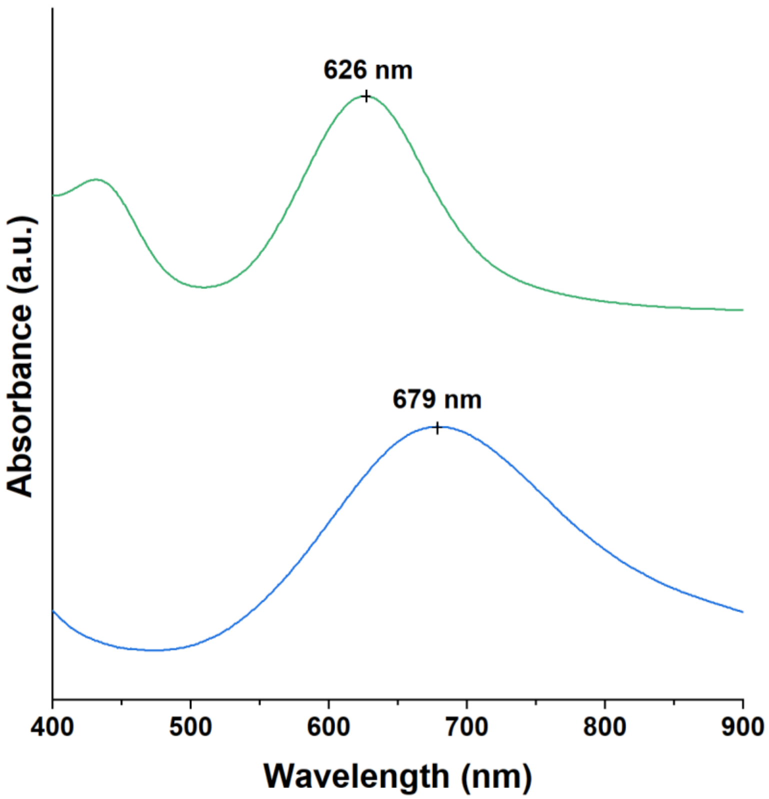

Supporting Figure 6. UV-Vis spectra of dissolved DUT-209 in $CHCl_3$. The adsorbed peaks regarding each metal paddlewheel cluster were observed in 679 nm (blue line) and 626 nm (green line) of UV-Vis spectra, respectively.

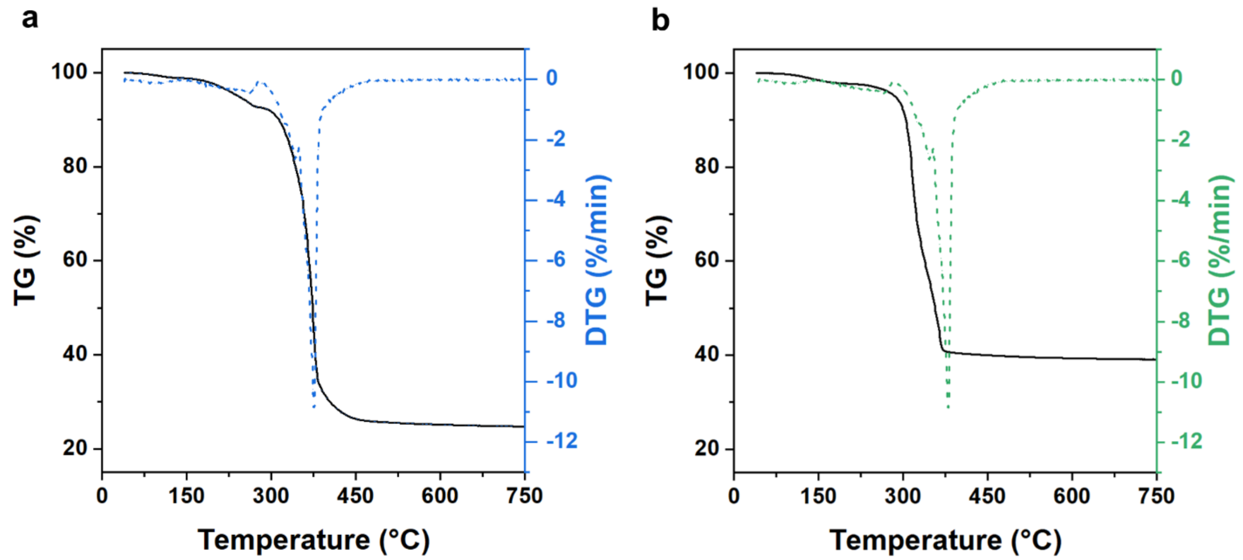

Supporting Figure 7. Thermogravimetric analysis of DUT-209. a, DUT-209(Cu) were decomposed at 370 °C. **b,** DUT-209(Rh) is slightly more stable than DUT-209(Cu).

4. Synthesis of 1,5-Bis[2-methyl-5-(4-pyridyl)-3-thienyl]cyclopentane (BPMTC) pillar

BPMTC, as the *N*-donor bidentate ligand based on diarylethene photochromic moiety, was synthesized by modified recipes.[22–24] The synthetic process of five steps is followed by Fig. S8.

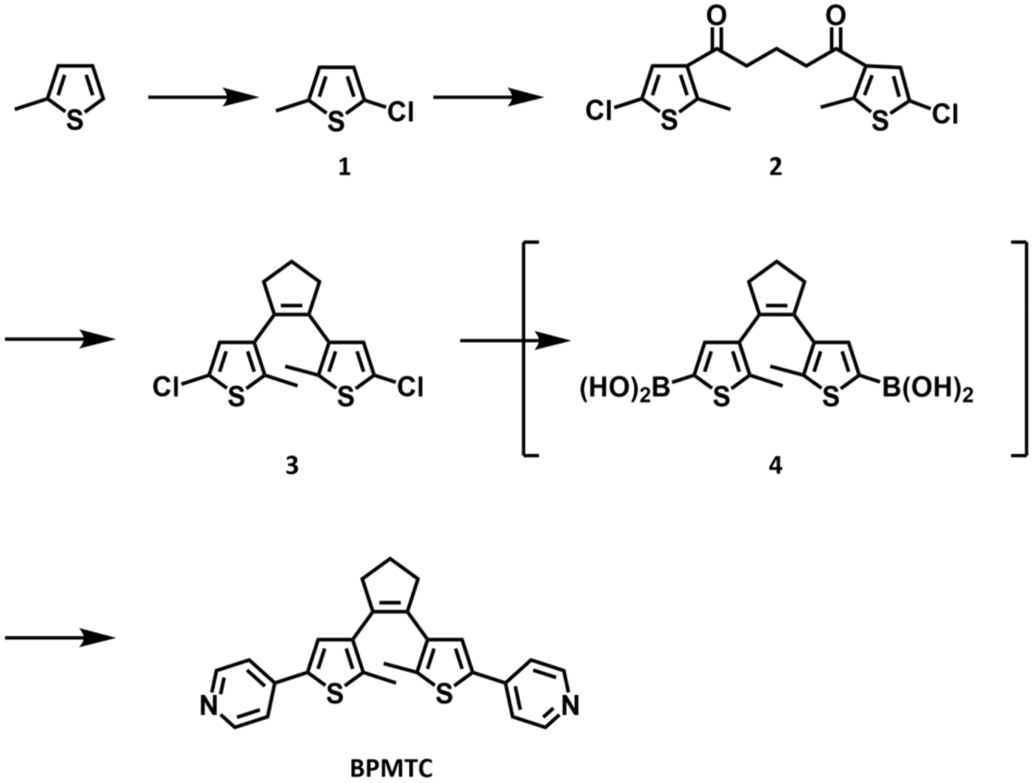

Supporting Figure 8. Synthetic process of BMPTC pillar.

4.1 Synthesis of 2-Chloro-5-methylthiophene, compound 1. 2-Methylthiophene (50 mL, 0.50 mol) and N-chlorosuccinimide (76 g, 0.50 mol) were added to a mixed solution of benzene (200 mL) and acetic acid (200 mL). The suspension was stirred for 30 minutes at room temperature, after then, refluxed for 1 hour. The yellow solution was cooled down and poured into a 3 M aq. NaOH solution (150 mL). The organic phase was washed with a 3 M aq. NaOH solution (3 × 150 mL), filtered on Na_2SO_4. After evaporation of residual solvents, the obtained yellow solution was purified by vacuum distillation at 55 °C to a colorless liquid. (44.41 g, 67 %). ^{1}H NMR (300 MHz, $CDCl_3$): δ = 6.53 (d, *J* = 6.54 Hz, 1H), 6.36 (d, *J* = 6.36 Hz, 2H), 2.25 (s, 3H) ppm.

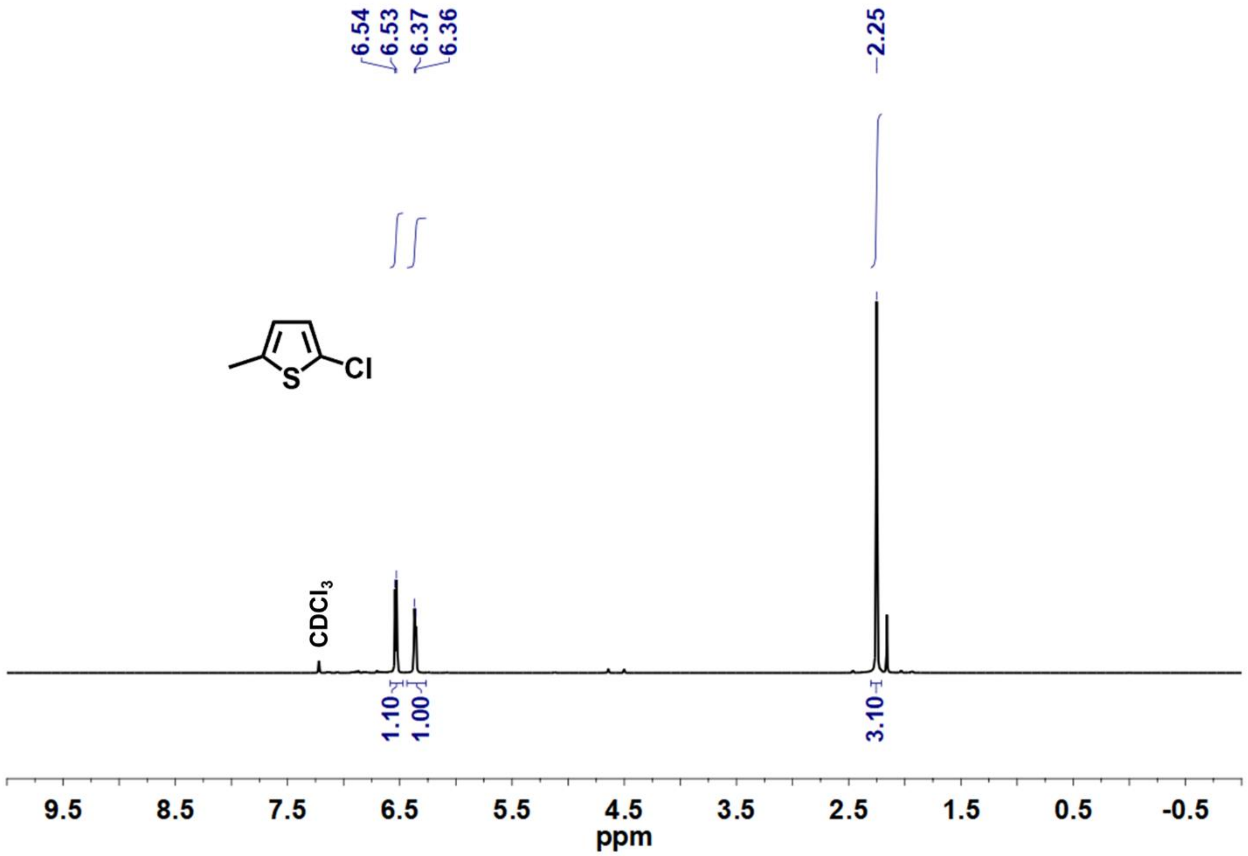

Supporting Figure 9. ^{1}H NMR spectrum of compound **1**.

4.2 Synthesis of 1,5-Bis(5-chloro-2-methylthien-3-yl)pentane-1,5-dione, compound 2. Half portion of anhydrous $AlCl_3$ (24g, 0.18 mol) was added in a mixed solution of an ice-cooled solution of compound **1** (16.15 mL, 0.15 mol), glutaryl dichloride (12.5 g, 73.7 mmol) and CS_2 (150 mL) under vigorous stirring at 0 °C. After another half portion of $AlCl_3$ was added, the mixture was stirred for 2 hours at room temperature. After then, the ice-cold water (50 mL) was carefully added to the mixture and the water layer was extracted with diethyl ether (3 × 75 mL). The combined organic phases were washed with water (50 mL), filtered on Na_2SO_4, and the solvent was evaporated. The obtained tar can be purified by recrystallization ($CHCl_3$/MeOH) (3.05 g, 5.7 %). ^{1}H NMR (300 MHz, $CDCl_3$): δ = 7.11 (s, 1H), 2.79 (t, *J* = 6.9 Hz, 2H), 2.59 (s, 3H), 1.99 (p, *J* = 6.9 Hz, 1H) ppm.

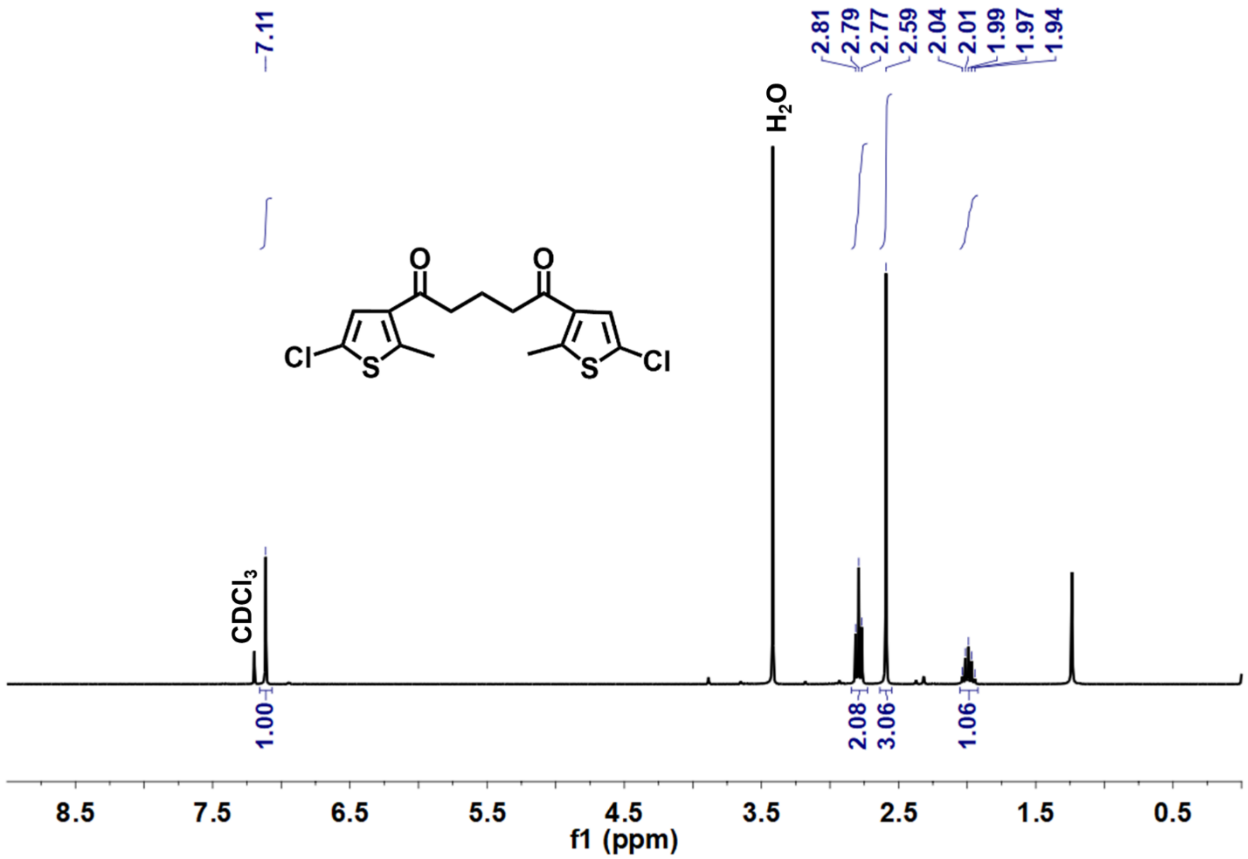

Supporting Figure 10. ^{1}H NMR spectrum of compound **2**.

4.3 Synthesis of 1,5-Bis(5-chloro-2-methylthien-3-yl)cyclopentene, compound 3. $TiCl_3(THF)_3$ (5.12 g, 13.81 mmol), Zn dust (1.85 g, 28.2 mmol) and THF (66 mL) was stirred under nitrogen for 45 minutes at 65 °C. After the mixture cooled down to 0 °C, compound **2** (2.49 g, 6.89 mmol) was added in this mixture and heated to 65 °C for 4 hours. The mixture, to which a few drops of saturated aqueous K_2CO_3 solution were added, was filtered over Celite and washed with EtOAc. The filtrate was dried over anhydrous $MgSO_4$ and the solvent was evaporated. The crude product was purified by column chromatography using hexanes. (2.01 g, 88.5 %). ^{1}H NMR (300 MHz, $CDCl_3$): δ = 6.50 (s, 1H), 2.64 (t, *J* = 7.5 Hz, 2H), 1.95 (dq, *J* = 14.9, 7.5 Hz, 1H), 1.81 (s, 3H) ppm.

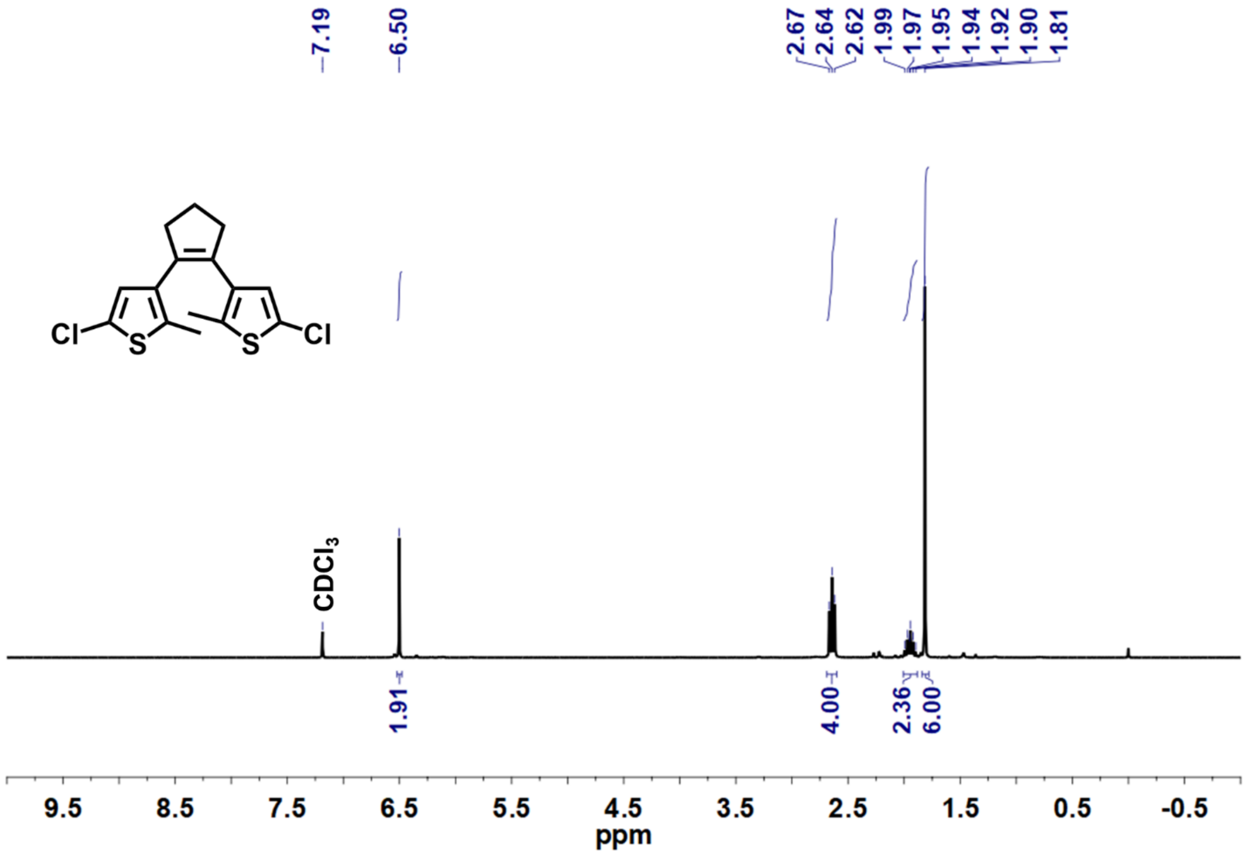

Supporting Figure 11. ^{1}H NMR spectrum of compound **3**.

4.4 Synthesis of BPMTC. Compound **3** (3.03 g, 9.21 mmol) and dry THF (37.3 mL) were added under Ar atmosphere. The mixture was cooled with an ice bath and 2.5 M n-BuLi (8.09 mL, 87.4 mmol) was slowly added and stirred for 30 minutes. Tris-n-butylborate (7.45 mL, 26.3 mmol) was added and warmed up to room temperature, and additionally stirred for 1 hr. At the same time, $Pd(PPh_3)_4$ (620 mg, 0.53 mmol) was suspended in THF (31 mL) in another batch and the mixture was heated at 70 °C for 30 minutes. Aqueous 2.5 M K_2CO_3 (37.2 mL), ethylene glycol (12 drops), 4-bromopyridine hydrochloride (3.92 g, 29.0 mmol) were added. This batch was slowly added to the first batch, Compound **4**. The mixture was heated at 70 °C overnight. The mixture was cooled to RT and added water. The mixture was extracted with diethyl ether and the crude solution was dried over anhydrous $MgSO_4$. The solvent was evaporated under reduced pressure. The crude product was purified by column chromatography (silica, iso-hexene/ethyl acetate). ^{1}H NMR (300 MHz, $CDCl_3$): δ = 8.43 (dd, *J* = 4.6, 1.6 Hz, 4H), 7.24 (dd, *J* = 4.6, 1.6 Hz, 4H), 7.13 (s, 2H), 2.76 (t, *J* = 7.5 Hz, 4H), 2.09-1.97 (m, 1H), 1.93 (s, 6H) ppm. ^{13}C NMR (100 MHz, $CDCl_3$): δ = 150.27, 141.29, 137.30, 137.10, 136.66, 134.81, 126.31, 119.26, 38.46, 30.92, 22.99, 14.65 ppm. HRMS (HR-ESI-MS) cal. for $[M+H]^+$ *m/z* 414.58 g/mol, found *m/z* 415.13 g/mol.

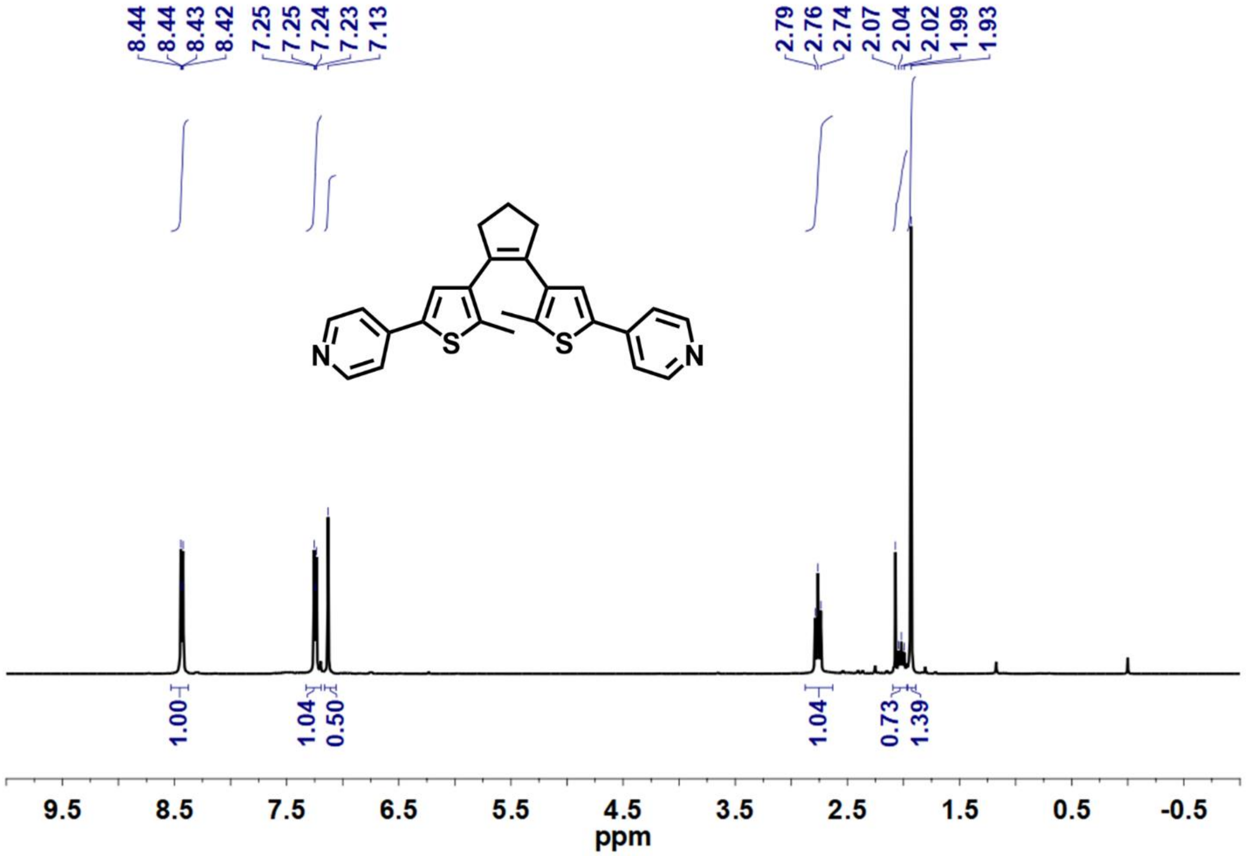

Supporting Figure 12. ^{1}H NMR spectrum of **BPMTC**.

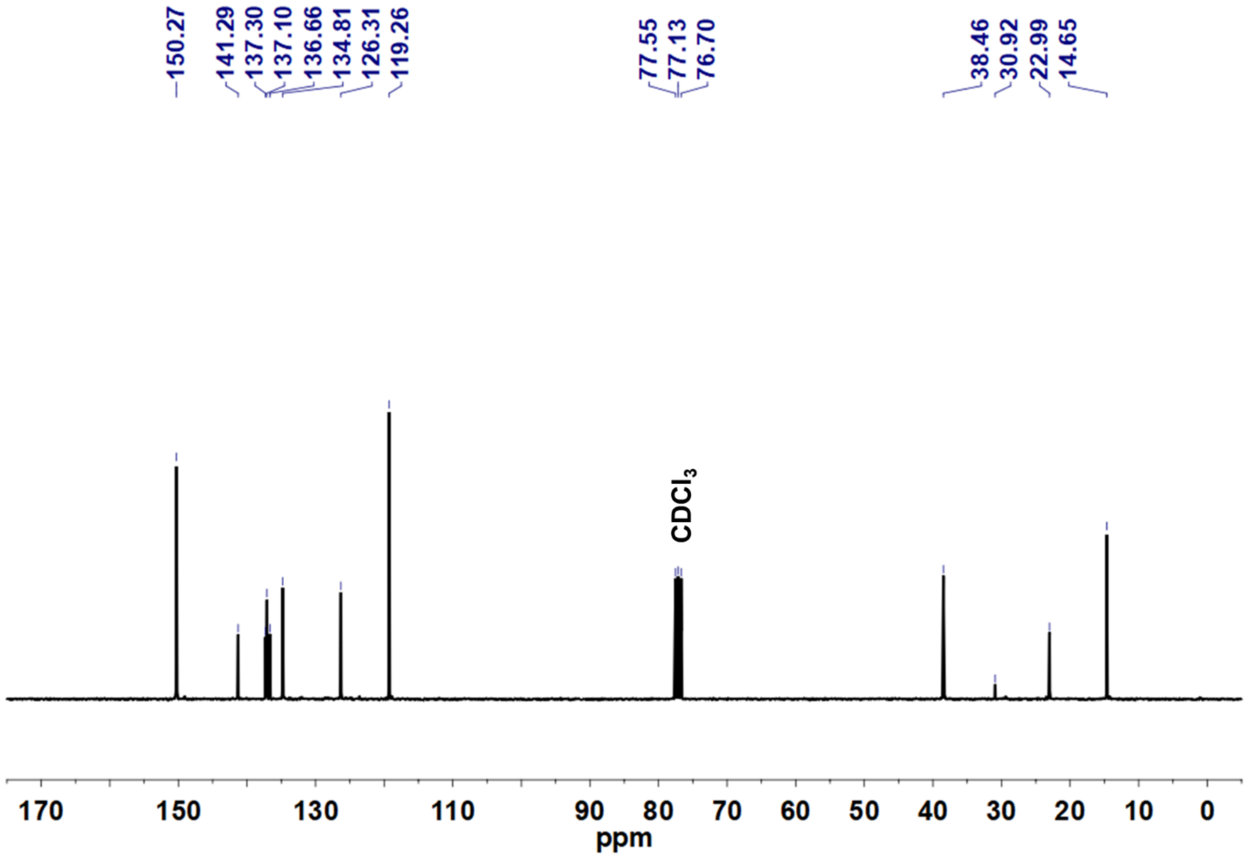

Supporting Figure 13. ^{13}C NMR spectrum of **BPMTC**.

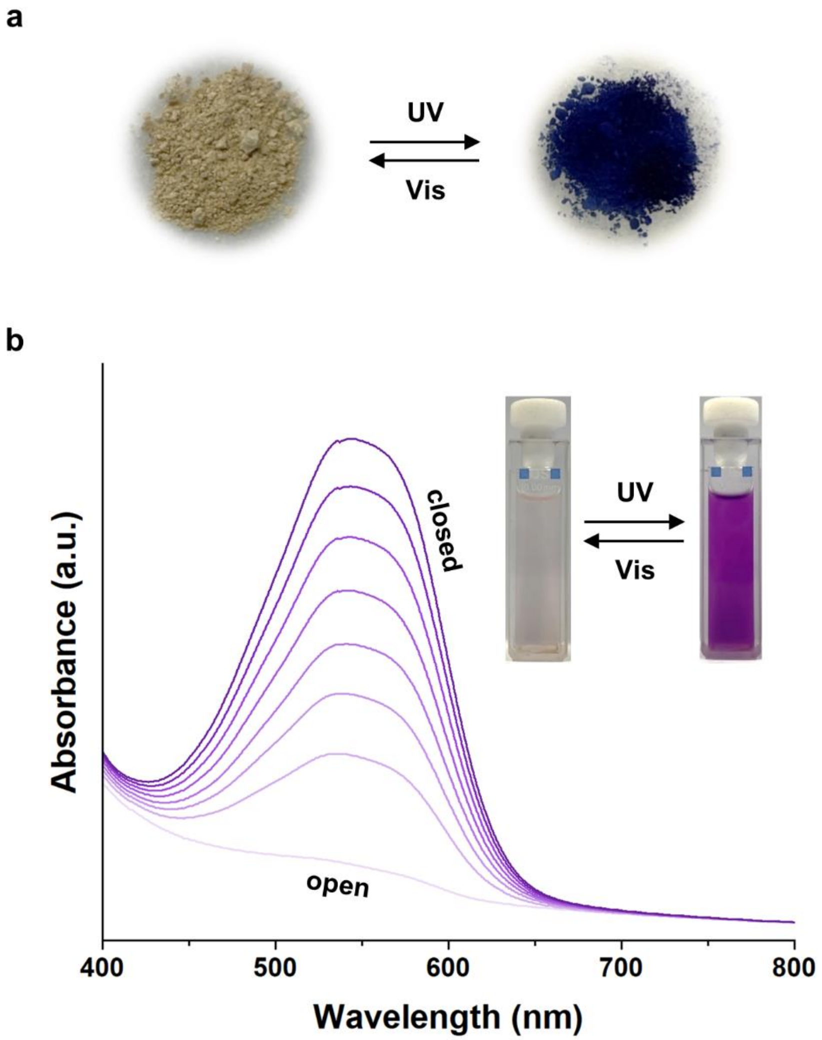

Supporting Figure 14. Reversible color change and photoisomerization of BPMTC powder and solution obtained after irradiation under 365 nm and 550 nm. a, The color change in solid-state. **b,** Light-induced *in-situ* liquid UV-Vis spectra of BPMTC solution in $CHCl_3$. The intensive purple color of the *c*-form turned transparent (*o*-form), as seem from the decrease in absorbance of the peak.

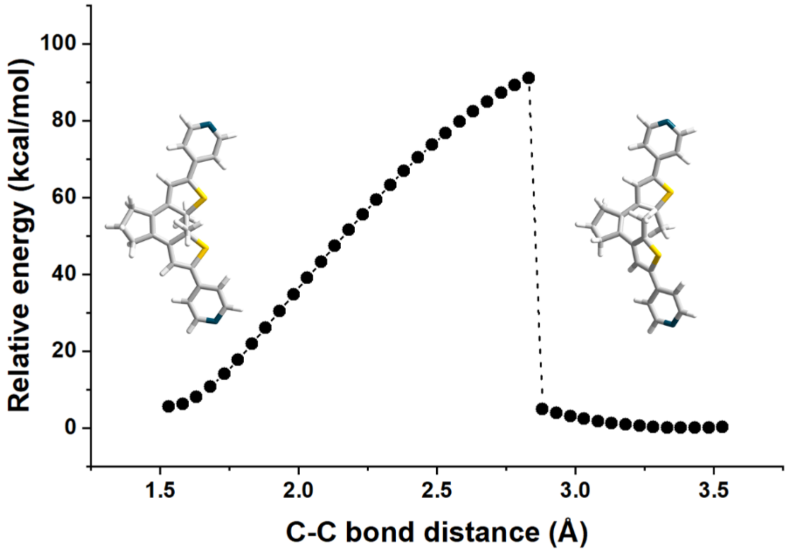

Supporting Figure 15. Potential energy profile for the mutual conversion between the *c*-form and the *o*-form(*ap*). The data points were obtained by performing a relaxed potential energy scan calculation with respect to the newly formed (or, cleaved) C-C bond (between the two carbon atoms bearing the two methyl groups attached to the two thiophene rings) distance at the PBE0-D3(BJ)/def2-SVP level of theory. The *c*-form was chosen as the starting point for the scan calculation. The barrier was calculated from the difference in energy between the top point and the *o*-form(*ap*) and amounts to 91.1 kcal/mol.

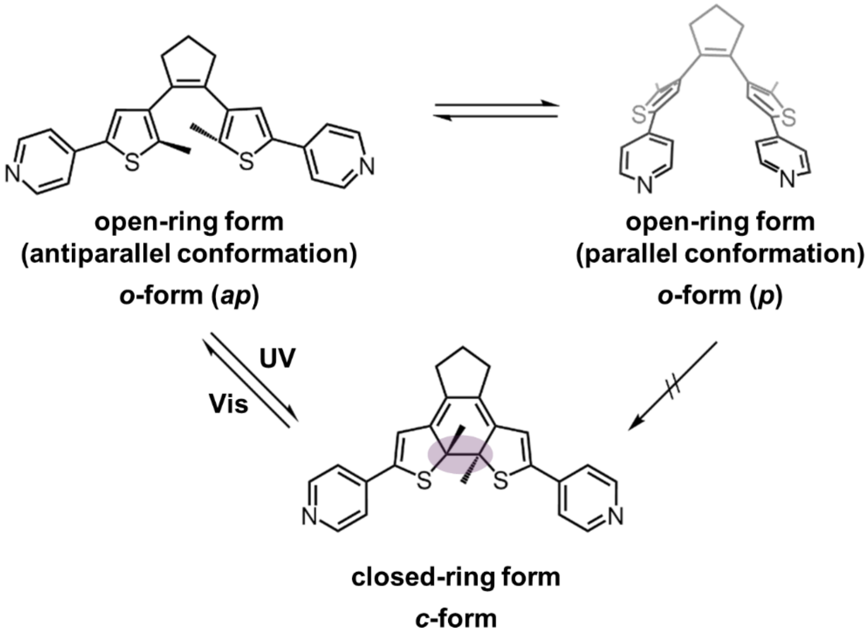

Supporting Figure 16. Schematic representation of reversible photoisomerization and conformation of BPMTC ligand. The open-ring form with antiparallel conformation; *o*-form(*ap*) is photoisomerized to closed-ring form; *c*-form under UV light and turning back to *o*-form(*ap*) under visible light. The *o*-form has different types of conformations which are *o*-form(*ap*) and parallel; *o*-form(*p*).

5. Supramolecular materials via assembly of MOPs and *N*-donor bidentate ligands.

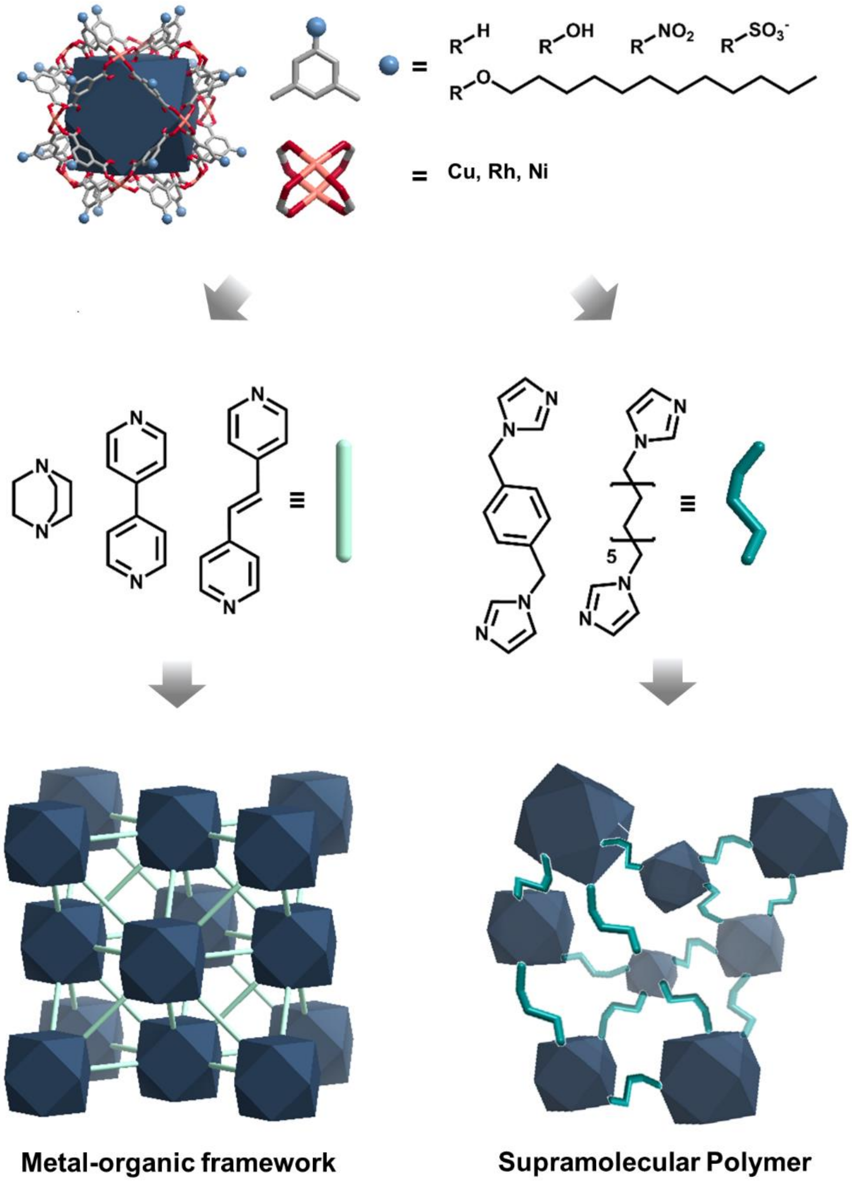

Supporting Figure 17. Two types of compounds via assembly of MOPs as SBBs and *N*-donor bidentate ligands. The crystalline frameworks were implemented using liner bidentate ligands with length from 2.7 to 11.5 Å. The supramolecular polymers as gels were built from non-linear bidentate ligands and functionalized MOPs.

6. Characterization of MOFs, DUT-210(Cu) and DUT-210(Rh)

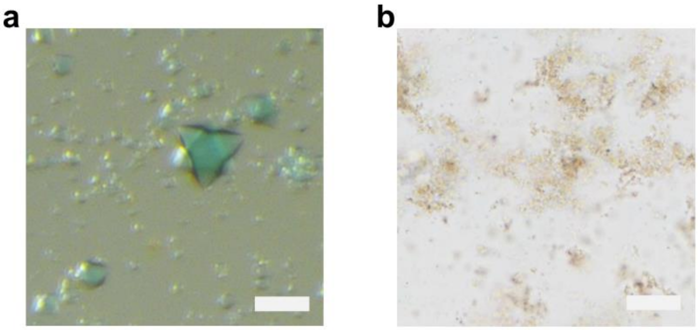

Supporting Figure 18. Optical microscopic images of synthesized DUT-210 (scale bar: 50 µm). a, DUT-210(Cu) crystals represent an octahedral shape and pale blue. **b,** DUT-210(Rh) crystals show red color.

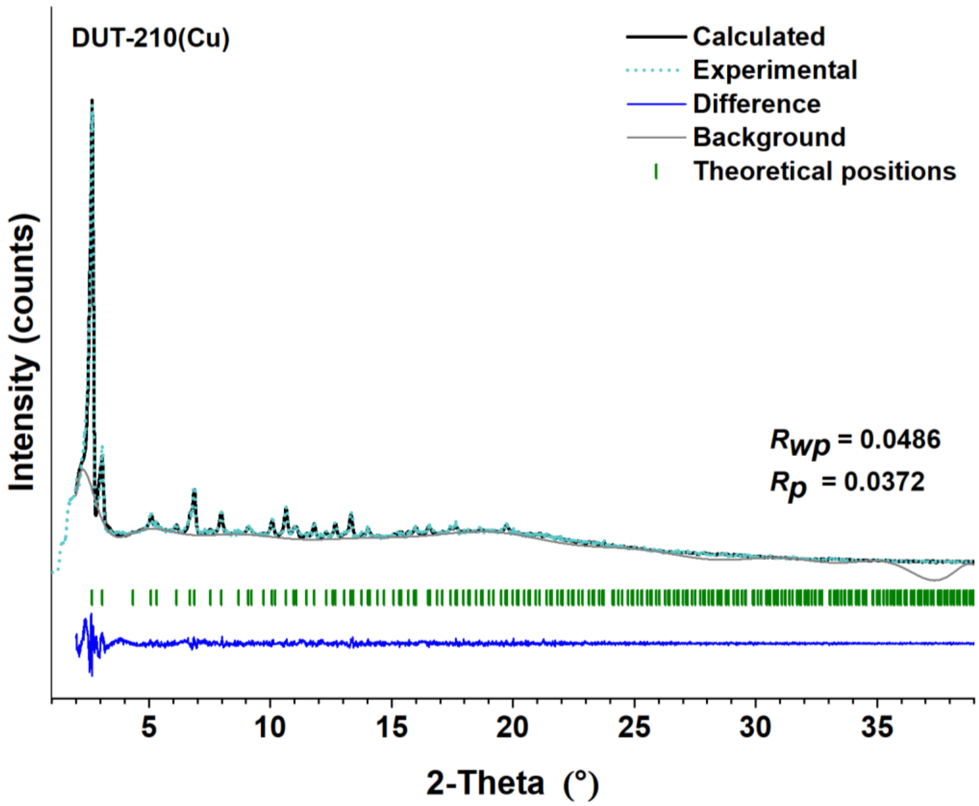

Supporting Figure 19. Pawley refinement of the PXRD patterns, measured on acetone solution of DUT-210(Cu). Profile data: cubic, *Fm*-3*m*, *a* = 57.508(3) Å, *λ* = 1.54059 Å, profile function: Thompson-Cox-Hastings, U = 0.05819, V = -0.01587, W = 0.00490, X = 0.31817, Y = 0.09338; asymmetry correction: P1 = -0.06228, P2 = -0.01094, P3 = 0.00676, P4 = 0.00162.

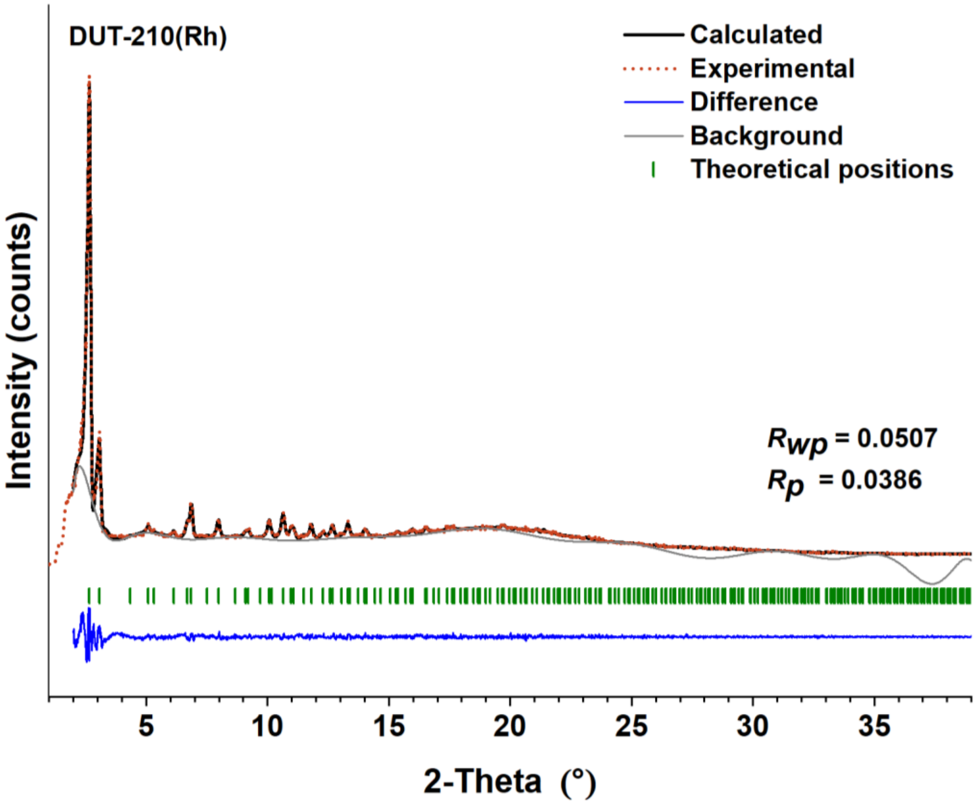

Supporting Figure 20. Pawley refinement of the PXRD patterns, measured on acetone solution of DUT-210(Rh). Profile data: cubic, *Fm*-3*m*, *a* = 57.5553(22) Å, λ = 1.54059 Å, profile function: Thompson-Cox-Hastings, U = 0.18880, V = -0.02308, W = 0.00446, X = 0.42470, Y = 0.10477; asymmetry correction: P1 = -0.05807, P2 = -0.01021, P3 = 0.00687, P4 = 0.00166.

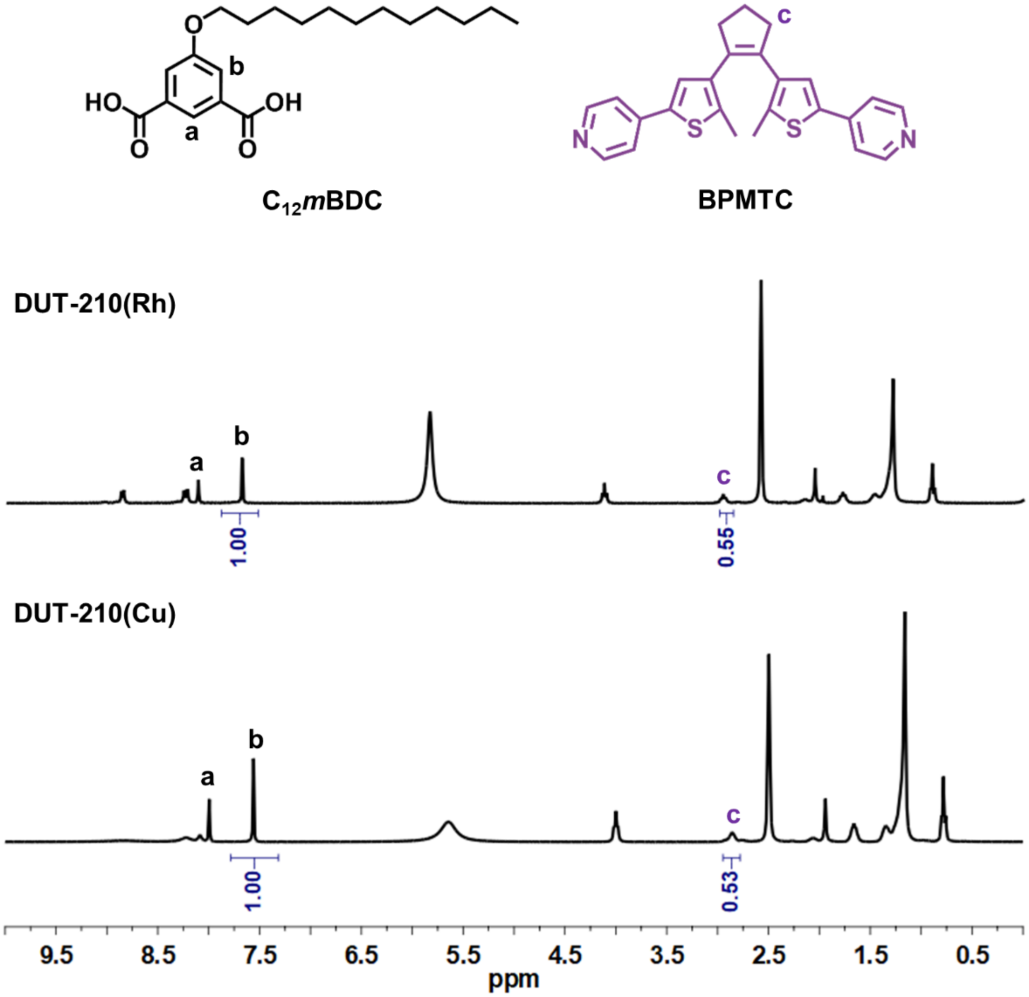

Supporting Figure 21. ^{1}H NMR spectra of dried DUT-210 after acid digestion. The samples were digested using a mixture of 0.5 mL of DMSO-d_6 and 0.1 mL of diluted DCl (0.5 mL of DMSO-d_6 and 100 μL of 35 % DCl in D_2O). The prepared samples were sonicated for about 1 hour and then heated in an oven at 100 °C. Two peaks of H_2O and DMSO-d_6 represent ~5.5 ppm and 2.50 ppm, respectively. The theoretical ratio of $C_{12}m$BDC to BPMTC was 4.0 to 1.0 in the framework.

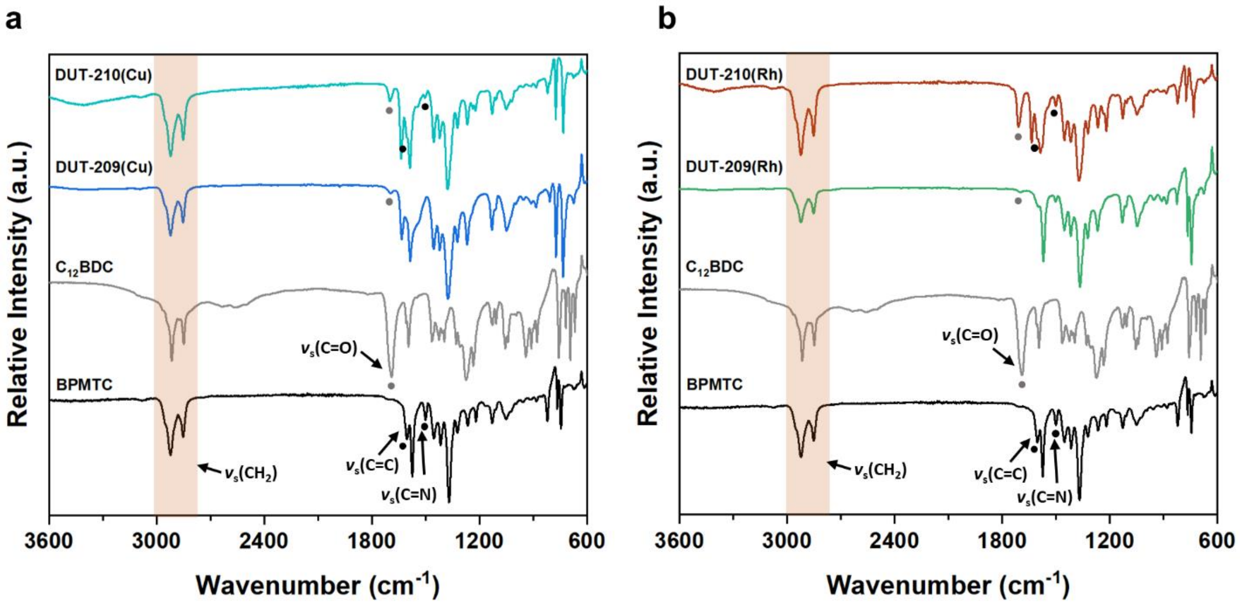

Supporting Figure 22. FT-IR spectra of the MOP units and photochromic MOFs with their structural components. a, Comparison of the FT-IR spectra of Cu-based materials; DUT-209(Cu) and DUT-210(Cu) with C_{12}*m*BDC and BPMTC. **b,** Comparison of the Rh-based materials; DUT-209(Rh) and DUT-210(Rh) with C_{12}*m*BDC and BPMTC.

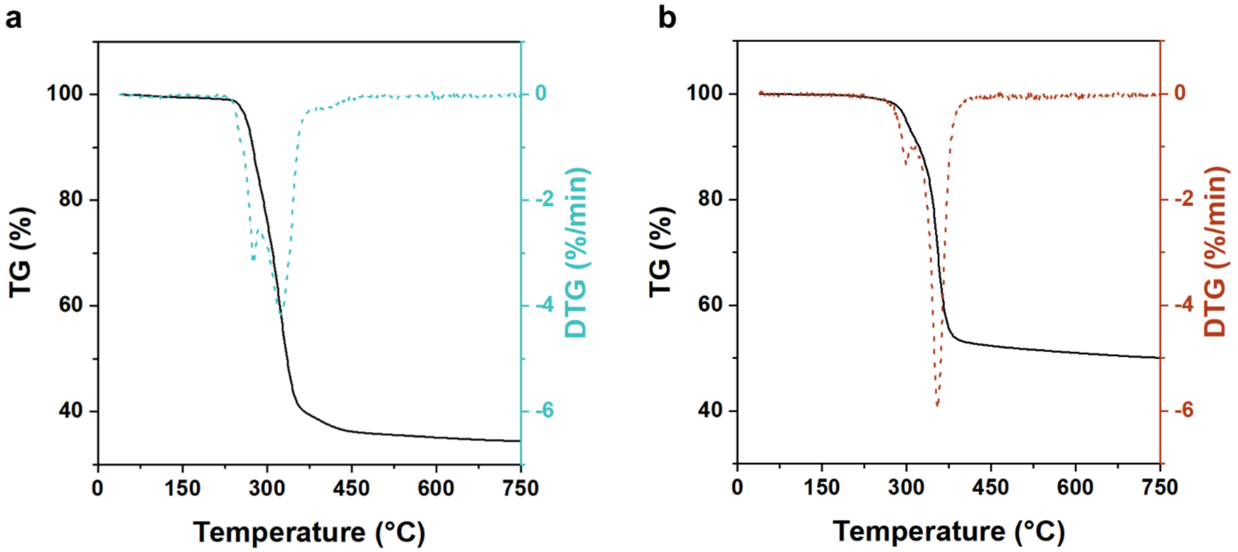

Supporting Figure 23. Thermogravimetric analysis of DUT-210. a, DUT-210(Cu) was decomposed at 350 °C, while DUT-209(Cu) was decomposed at 370 °C. **b,** Thermostability of DUT-209(Rh) and DUT-210(Rh) confirmed in the same temperature range. Both of the structures were decomposed at 370 °C.

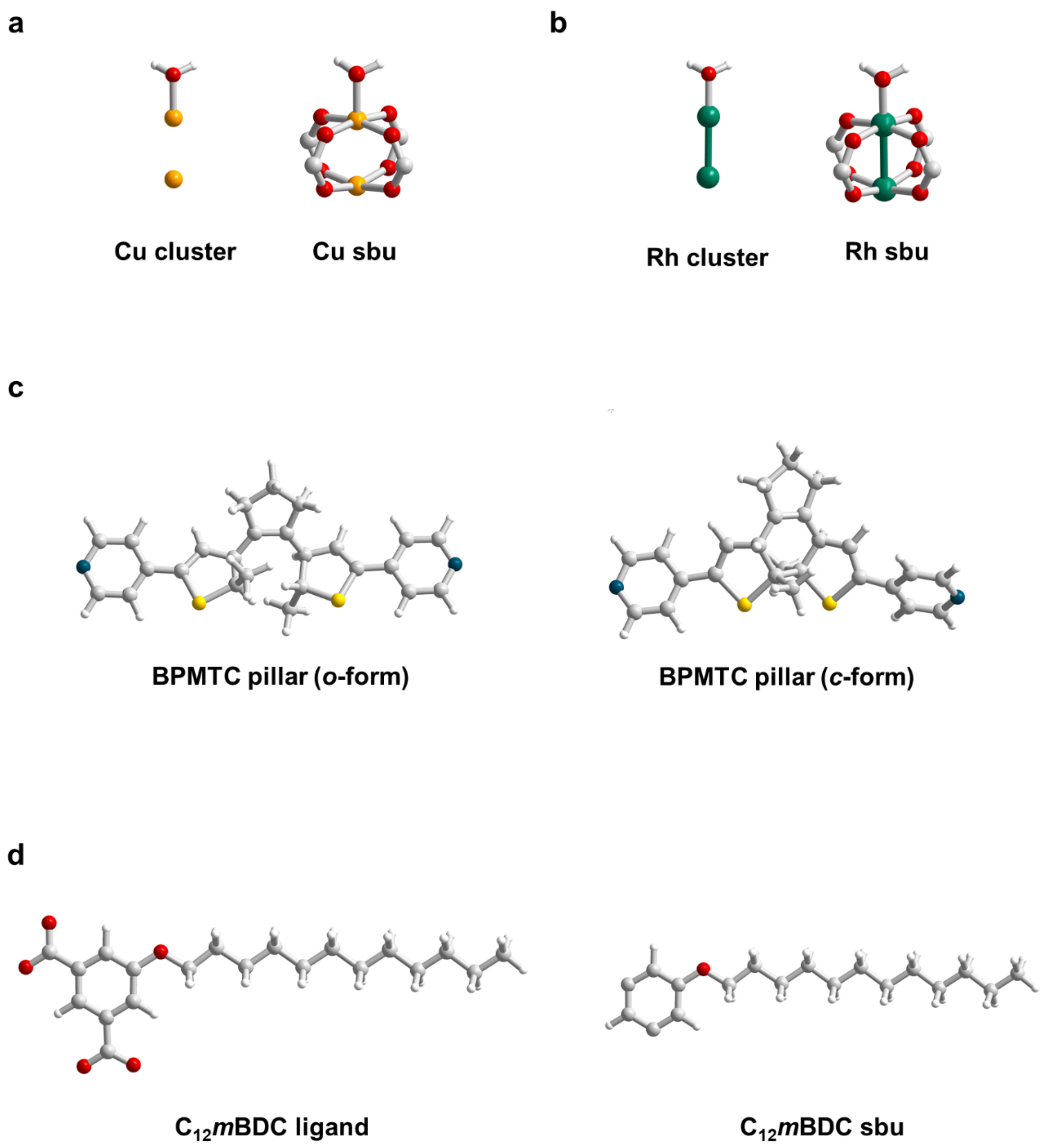

Supporting Figure 24. Building units obtained from deconstructing each MOF using mofstructure Python package. Metal cluster and SBU for **a,** Cu and **b,** Rh metals. **c,** Open and closed form representation of BPMTC pillar. **d,** Ligand and SBU form of C_{12}*m*BDC found in DUT-210. These structures both represent the ligand and organic sbu as deconstructed from the mofstructure Python package.

Supporting Table 1. Bond dissociation enthalpy for one formular unit of DUT-210 computed at GFN-xTB level of theory.

DUT-210	Ligands and metal clusters (kcal/mol)			Secondary building units (kcal/mol)		
Configuration	Cu	Rh	Cu → Rh	Cu	Rh	Cu → Rh
o-form (*ap*)	-111.38	-127.84	-16.45	-118.54	-127.35	-8.80
c-form	-109.92	-126.58	-16.67	-117.41	-127.09	-9.68
o-form (*ap*) → *c*-form	1.46	1.26	-0.22	1.13	0.26	-0.88

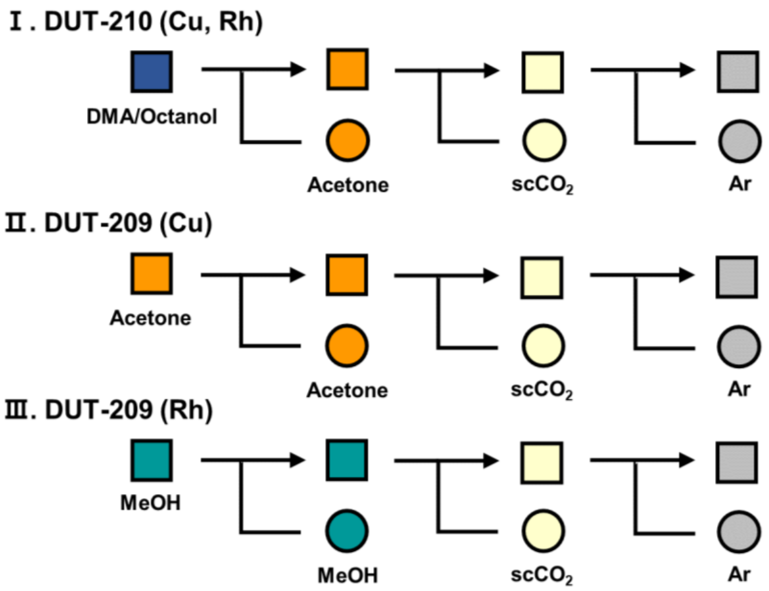

Supporting Figure 25. Symbolic representation of the DUT-209 and DUT-210 for activation. All of the samples were washed two times using each mother liquid. DUT-210 (Cu and Rh) and DUT-209(Cu) were washed four times for 2 days using acetone. DUT-209(Rh) was washed using MeOH, which was the recrystallization solvent. Supercritical CO_2 drying was conducted for 3 days and the chamber was heated up at 37 °C, maintaining the pressure of 100 kPa in the chamber. The heated chamber maintained for 1 hour and CO_2 slowly released and then Ar gas was refilled in the camber and flushed from the CO_2.

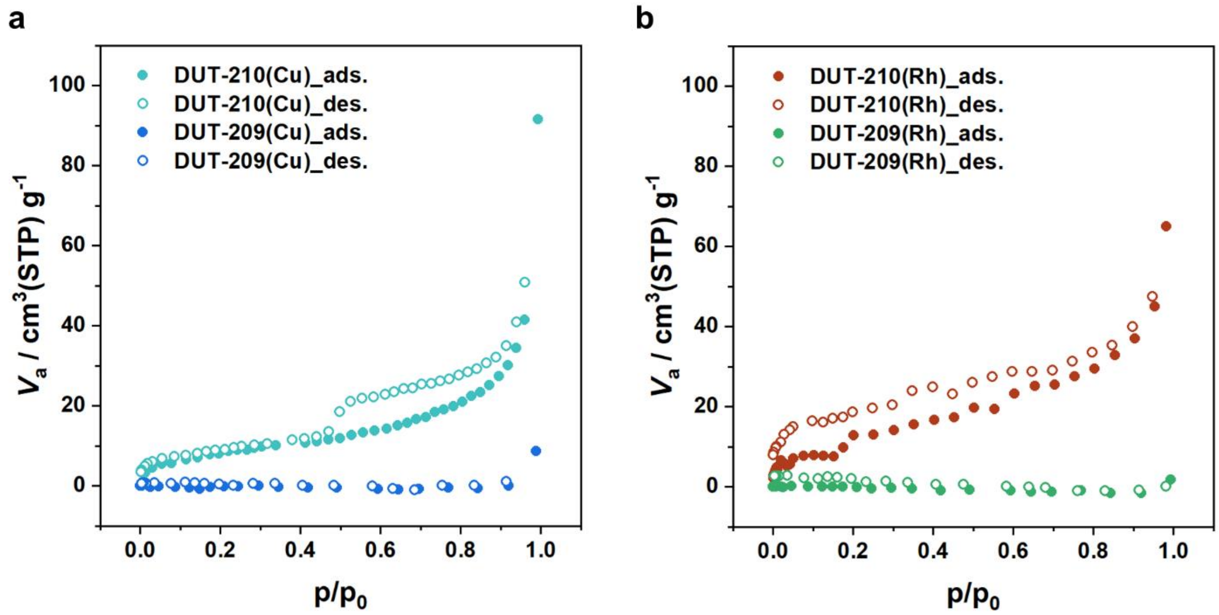

Supporting Figure 26. Nitrogen physisorption at 77 K on **a,** DUT-209 (Cu) and DUT-210(Cu)_*o*-form, **b,** DUT-209(Rh) and DUT-210(Rh)_*o*-form, respectively.

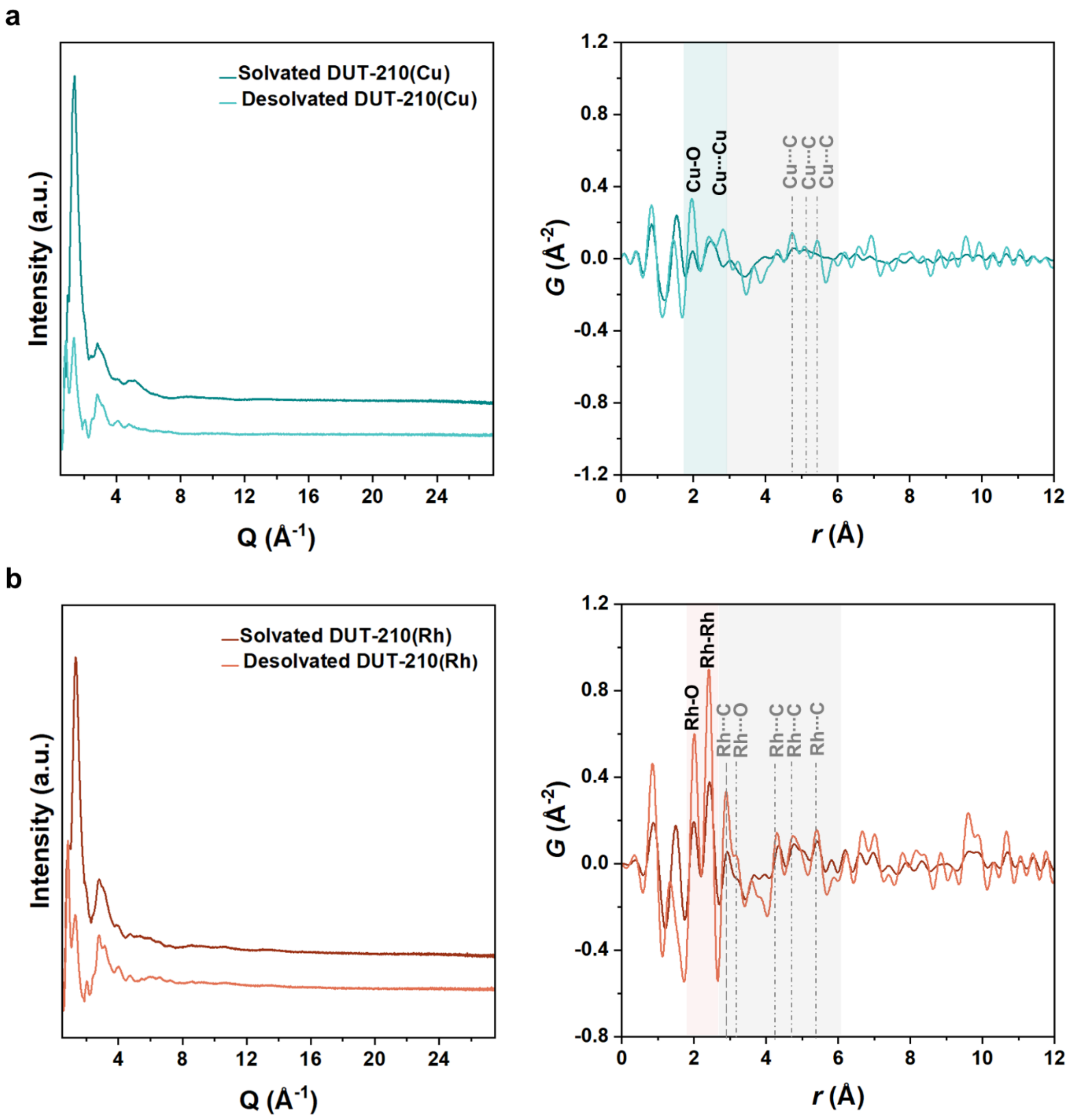

Supporting Figure 27. Pair Distribution Function data of solvated and desolvated DUT-210. G(r) curve of solvated and desolvated for **a,** DUT-210(Cu) and **b,** DUT-210(Rh).

7. Photochromic properties of DUT-210

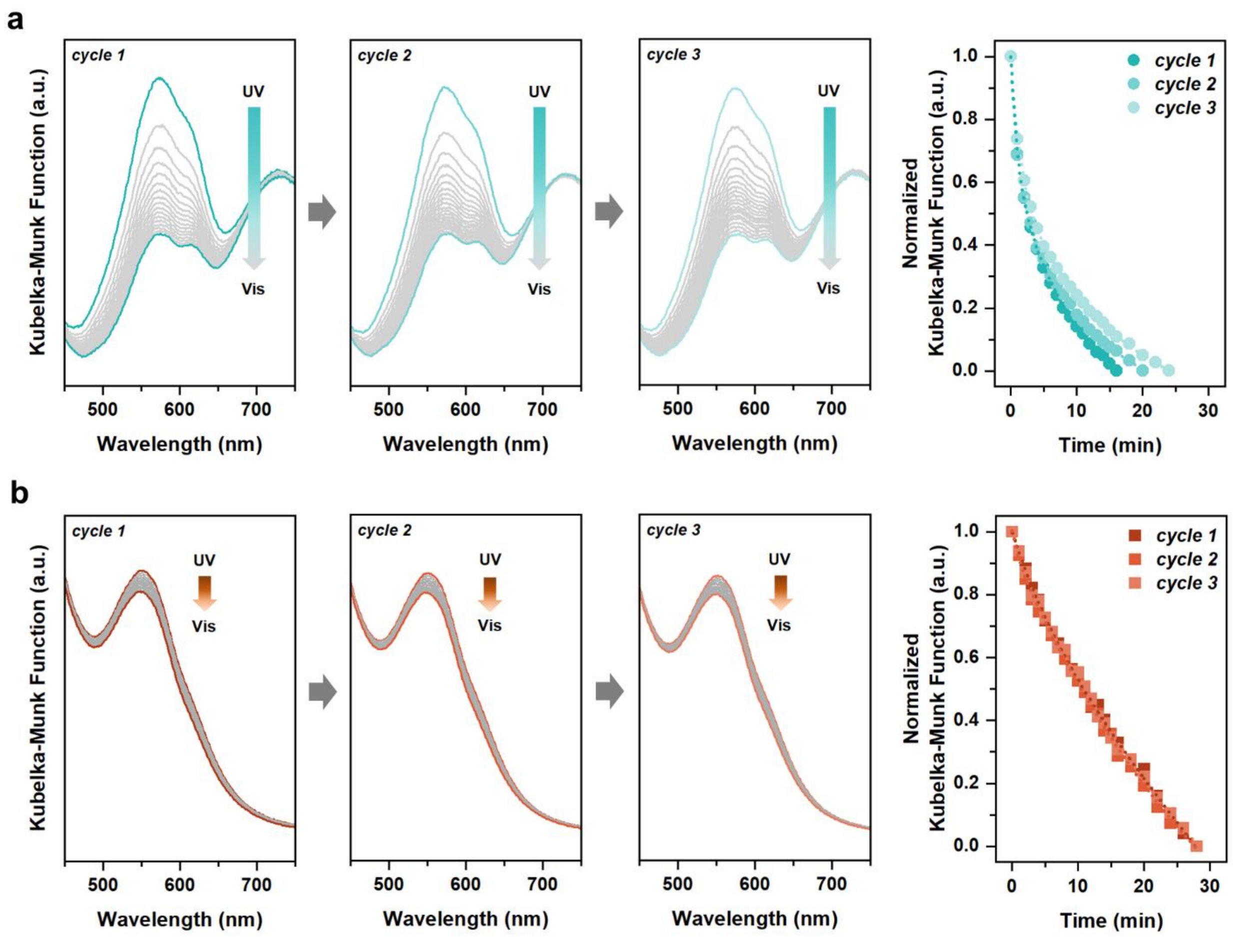

Supporting Figure 28. Time-resolved diffuse reflectance UV-Vis spectra upon irradiation. a, DUT-210(Cu), and **b,** DUT-210(Rh).

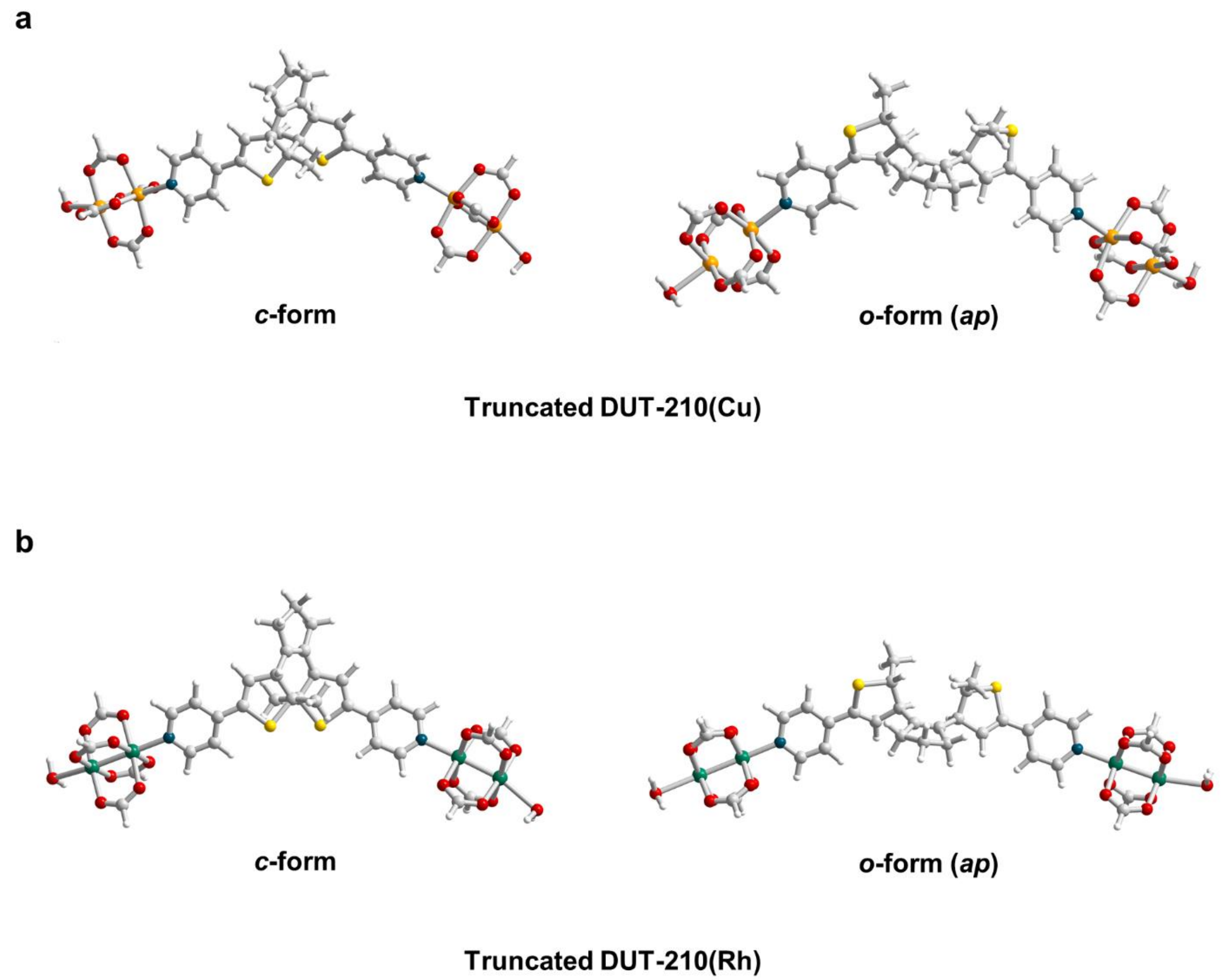

Supporting Figure 29. The *c*-form and *o*-form (*ap*) of truncated fragments of a, DUT-210(Cu) and b, DUT-210(Rh). In this truncated model, the C_{12}*m*BDC ligand was excluded based on the tenets that it does not have a major contribution to the overall electronic property of the system. Consequently, it was substituted with a hydrogen atom to ensure neutrality.

Supporting Table 2. Selected electrostatic potential of the connecting atoms in the truncated model of DUT-210 computed at PBE-D3/TZP of theory. Relativity was evaluated using spin-orbit coupling.

Atoms	DUT-210(Cu)		DUT-210(Rh)	
	***o*-form (*ap*) (a.u.)**	***c*-form (a.u.)**	***o*-form (*ap*) (a.u.)**	***c*-form (a.u.)**
Cu	137.308 / 137.32	137.308 / 137.321	-	-
Rh	-	-	257.356 / 257.328	257.356 / 257.327
N	18.317 / 18.316	18.317 / 18.317	18.30 / 18.298	18.299 / 18.299

Supporting Table 3. Metal-nitrogen (M-N) and metal-metal (M-M) bond lengths of open and closed configuration truncated DUT-210 computed at PBE-D3/TZP of theory. Relativity was evaluated using spin-orbit coupling. These bond lengths are all computed in the electronic ground state.

**Bonds length (Å)**	**GS-DUT-210(Cu)**		**GS-DUT-210(Rh)**	
	***o*-form (*ap*)**	***c*-form**	***o*-form (*ap*)**	***c*-form**
M-N	2.211	2.205	2.17	2.172
M-M	2.631	2.623	2.442	2.44
key	M=Cu		M=Rh	

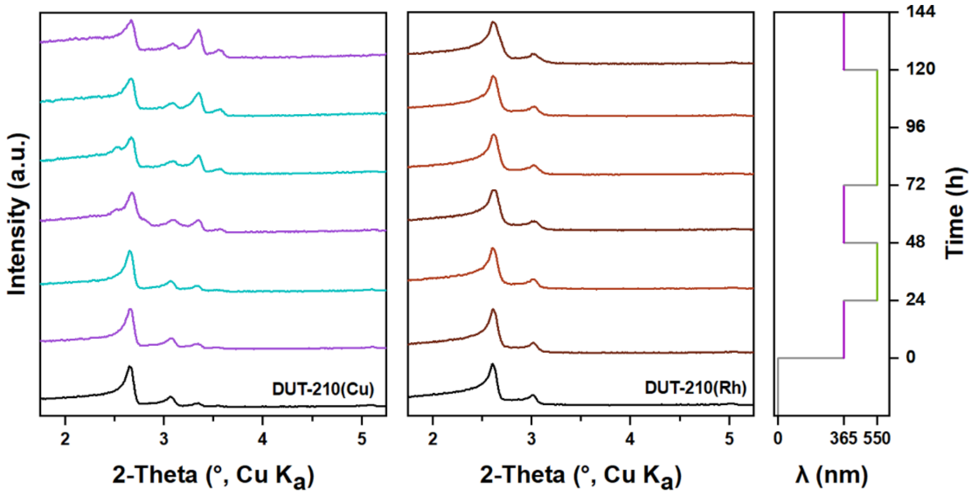

Supporting Figure 30. Photochromic structural transformation. *In situ*-PXRD data of DUT-210 series under the light.

Supporting Table 4. Ground and excited states energies of truncated DUT-210 (Cu) computed at PBE-D3/TZP of theory. Relativity was evaluated using spin-orbit coupling.

**Energy**	**GS-DUT210(Cu)**		**ES-DUT-210(Cu)**	
	***o*-form (*ap*)**	***c*-form**	***o*-form (*ap*)**	***c*-form**
Absolute energy (Kcal/mol)	-13678.47	-13504.38	-13796.18	-13623.24
HOMO (eV)	-5.3742	-5.387	-5.3177	-5.3326
LUMO (eV)	-4.8325	-4.8275	-4.7651	-4.7713
Band gap (eV)	0.5417	0.5595	0.5526	0.5614
o-form (*ap*) → *c*-form (kcal/mol)	174.09	174.09	172.94	172.94
Ground state → Excited stats (kcal/mol)	-117.71	-118.86	-117.71	-118.86

Supporting Table 5. Ground and excited states energies of truncated DUT-210 (Rh) computed at PBE-D3/TZP of theory. Relativity was evaluated using spin-orbit coupling.

**Energy**	**GS-DUT210(Rh)**		**ES-DUT-210(Rh)**	
	***o*-form (*ap*)**	***c*-form**	***o*-form (*ap*)**	***c*-form**
Absolute energy (Kcal/mol)	-14012.56	-13838.26	-14142.72	-13969.56
HOMO (eV)	-4.5312	-4.558	-4.5013	-4.53
LUMO (eV)	-3.0249	-3.1013	-2.9986	-3.0816
Band gap (eV)	1.5063	1.4566	1.5028	1.4483
o-form (*ap*) → *c*-form (kcal/mol)	174.3	174.3	173.16	173.16
Ground state → Excited stats (kcal/mol)	-130.16	-131.3	-130.16	-131.3

8. Reference

1. Mueller, U.; Förster, R.; Hellmig, M.; Huschmann, F. U.; Kastner, A.; Malecki, P.; Pühringer, S.; Röwer, M.; Sparta, K.; Steffien, M.; Ühlein, M.; Wilk, P.; Weiss, M. S. The macromolecular crystallography beamlines at BESSY II of the Helmholtz-Zentrum Berlin: Current status and perspectives. *Eur. Phys. J. Plus* **2015**, *130*, 141.
2. Battye, T. G. G.; Kontogiannis, L.; Johnson, O.; Powell, H. R.; Leslie, A. G. W. iMOSFLM: a new graphical interface for diffraction-image processing with MOSFLM. *Acta Crystallogr., Sect. D: Biol. Crystallogr.* **2011**, *67*, 271–281.
3. Sparta, K. M.; Krug, M.; Heinemann, U.; Mueller, U.; Weiss, M. S. XDSAPP2.0. *J. Appl. Crystallogr.* **2016**, *49*, 1085–1092.
4. Sheldrick, G. Crystal structure refinement with SHELXL. *Acta Crystallogr., Sect. C: Struct. Chem.* **2015**, *71*, 3–8.
5. Ashiotis, G.; Deschildre, A.; Nawaz, Z.; Wright, J. P.; Karkoulis, D.; Picca, F. E.; Kieffer, J. The fast azimuthal integration Python library: pyFAI, *J. Appl. Cryst*. **2015**, *48*, 510–519.
6. Juhás, P.; Davis, T.; Farrow, C. L.; Billinge, S. J. L. PDFgetX3: a rapid and highly automatable program for processing powder diffraction data into total scattering pair distribution functions. *J. Appl. Cryst*. **2013**, *46*, 560–566.
7. Yang, X.; Juhas, P.; Farrow, C. L.; Billinge, S. J. L. xPDFsuite: an end-to-end software solution for high throughput pair distribution function transformation, visualization and analysis. arXiv:1402.3163 [cond-mat.mtrl-sci].
8. Furukawa, H.; Kim, J.; Plass, K. E.; Yaghi, O. M. Crystal structure, dissolution, and deposition of a 5 nm functionalized metal-organic great rhombicuboctahedron. *J. Am. Chem. Soc*. **2006**, *128*, 8398–8399.
9. Ghosh, A. C.; Legrand, A.; Rajapaksha, R.; Craig, G. A.; Sassoye, C.; Balázs, G.; Farrusseng, D.; Furukawa, S.; Canivet, J.; Wisser, F. M. Rhodium-based metal-organic polyhedral assemblies for selective CO_2 photoreduction. *J. Am. Chem. Soc*. **2022**, *144*, 8, 3626–3636.
10. Warzecha, E.; Berto, T. C.; Berry, J. F. Axial ligand coordination to the C-H amination catalyst $Rh_2(esp)_2$: A structural and spectroscopic study. *Inorg. Chem*. **2015**, *54*, 17, 8817–8824
11. Perdew, J. P.; Burke, K.; Ernzerhof, M. Generalized gradient approximation made simple. *Phys. Rev. Lett.* **1996**, *77*, 3865.
12. Adamo, C.; Barone, V. Toward reliable density functional methods without adjustable parameters: The PBE0 model. *J. Chem. Phys*. **1999**, *110*, 6158–6170.
13. Grimme, S.; Antony, J.; Ehrlich, S.; Krieg, H. A consistent and accurate ab initio parametrization of density functional dispersion correction (DFT-D) for the 94 elements H-Pu. *J. Chem. Phys*. **2010**, *132*, 154104.
14. Grimme, S.; Ehrlich, S.; Goerigk, L. Effect of the damping function in dispersion-corrected density functional theory. *J. Comput. Chem*. **2011**, *32*, 1456–1465.
15. Frisch, M. J.; Trucks, G. W.; Schlegel, H. B.; Scuseria, G. E.; Robb, M. A.; Cheeseman, J. R.; Scalmani, G.; Barone, V.; Petersson, G. A.; Nakatsuji, H. et al. *Gaussian 16 Revision A.03*. 2016, Gaussian Inc. Wallingford CT
16. Weigend, F.; Ahlrichs, R. Balanced basis sets of split valence, triple zeta valence and quadruple zeta valence quality for H to Rn: Design and assessment of accuracy. *Phys. Chem. Chem. Phys*. **2005**, *7*, 3297–3305.

17. Grimme, S.; Bannwarth, C.; Shushkov, P. A Robust and Accurate Tight-Binding Quantum Chemical Method for Structures, Vibrational Frequencies, and Noncovalent Interactions of Large Molecular Systems Parametrized for All spd-Block Elements (Z = 1–86***).*** *J. Chem. Theory Comput.* **2017***, 13*, 1989–2009.
18. Perdew, John P.; Burke, K.; Ernzerhof, M. Generalized Gradient Approximation Made Simple. *Rev. Lett*. **1996**, 78, 1396.
19. Ernest R. Davidson. The iterative calculation of a few of the lowest eigenvalues and corresponding eigenvectors of large real-symmetric matrices. *J. Comput. Phys.* **1975**, *17*, 87-94
20. Velde, G. T.; Bickelhaupt, F. M.; Baerends, E. J.; Fonseca Guerra, C.; Van Gisbergen, J. A.; Snijders, J. G.; Ziegler, T. Chemistry with ADF. *J. Comput. Chem*. **2001**, *22*, 931–967.
21. Broto-Ribas, A.; Gutiérrez, M. S.; Imaz, I.; Carné-Sánchez, A.; Gándara, F.; Juanhuix, J.; Maspoch, D. Synthesis of the two isomers of heteroleptic $Rh_{12}L_6L'_6$ metal-organic polyhedra by screening of complementary linkers. *Chem. Commun*., **2022**, *58*, 10480–10483.
22. Lucas, L. N.; de Jong, J. J. D.; van Esch, J. H.; Kellogg, R. M.; Feringa, B. L. Syntheses of dithienylcyclopentene optical molecular switches. *Eur. J. Org. Chem*. **2003**, 155–166.
23. Qin, B.; Yao, R.; Zhao, X.; Tian, H. Enhanced photochromism of 1,2-dithienylcyclopentene complexes with metal ion. *Org. Biomol. Chem*., **2003**, *1*, 2187–2191.
24. Park, J.; Feng, D.; Yuan, S.; Zhou, H.-C. Photochromic metal-organic frameworks: Reversible control of singlet oxygen generation. *Angew. Chem. Int. Ed*. **2015**, *54*, 430–435.
